# Comprehensive phylogenomic analyses re-write the evolution of parasitism within cynipoid wasps

**DOI:** 10.1186/s12862-020-01716-2

**Published:** 2020-11-23

**Authors:** Bonnie B. Blaimer, Dietrich Gotzek, Seán G. Brady, Matthew L. Buffington

**Affiliations:** 1 Center for Integrative Biodiversity Discovery, Museum für Naturkunde, Berlin, Germany; 2 National Museum of Natural History, Smithsonian Institution, Washington, DC USA; 3 North Carolina State University, Raleigh, NC USA; 4 Systematic Entomology Laboratory, ARS-USDA, C/O NMNH, Smithsonian Institution, Washington, DC USA

**Keywords:** Parasitoidism, Parasitism, Galling, Inquilinism, Ultraconserved elements, Phylogenomics, Cynipoidea, Cynipidae, Figitidae

## Abstract

**Background:**

Parasitoidism, a specialized life strategy in which a parasite eventually kills its host, is frequently found within the insect order Hymenoptera (wasps, ants and bees). A parasitoid lifestyle is one of two dominant life strategies within the hymenopteran superfamily Cynipoidea, with the other being an unusual plant-feeding behavior known as galling. Less commonly, cynipoid wasps exhibit inquilinism, a strategy where some species have adapted to usurp other species’ galls instead of inducing their own. Using a phylogenomic data set of ultraconserved elements from nearly all lineages of Cynipoidea, we here generate a robust phylogenetic framework and timescale to understand cynipoid systematics and the evolution of these life histories.

**Results:**

Our reconstructed evolutionary history for Cynipoidea differs considerably from previous hypotheses. Rooting our analyses with non-cynipoid outgroups, the Paraulacini, a group of inquilines, emerged as sister-group to the rest of Cynipoidea, rendering the gall wasp family Cynipidae paraphyletic. The families Ibaliidae and Liopteridae, long considered archaic and early-branching parasitoid lineages, were found nested well within the Cynipoidea as sister-group to the parasitoid Figitidae. Cynipoidea originated in the early Jurassic around 190 Ma. Either inquilinism or parasitoidism is suggested as the ancestral and dominant strategy throughout the early evolution of cynipoids, depending on whether a simple (three states: parasitoidism, inquilinism and galling) or more complex (seven states: parasitoidism, inquilinism and galling split by host use) model is employed.

**Conclusions:**

Our study has significant impact on understanding cynipoid evolution and highlights the importance of adequate outgroup sampling. We discuss the evolutionary timescale of the superfamily in relation to their insect hosts and host plants, and outline how phytophagous galling behavior may have evolved from entomophagous, parasitoid cynipoids. Our study has established the framework for further physiological and comparative genomic work between gall-making, inquiline and parasitoid lineages, which could also have significant implications for the evolution of diverse life histories in other Hymenoptera.

## Background

One of the most intriguing biological interactions between organisms is parasitism, a life history strategy in which one partner, the parasite, has a detrimental effect on another, the host [1]. Parasitism occurs broadly across the tree of life, with a multitude of variations in the specific interactions between parasite and host. A notable diversity of parasitic lifestyles exists within the insects, such as ectoparasitism in lice and fleas [2], cleptoparasitism in bees [3], or social parasitism in ants [4]. A specialized form of parasitism exclusive to insects is parasitoidism. In this life history strategy, the adult lays eggs in the immature stages of a host arthropod (typically insects), and the developing immature parasitoid feeds on and ultimately kills its host (necessitating the term parasitoid, and not simply ‘parasite’). The Hymenoptera, best known for the ants, bees, and stinging wasps, also include many members with parasitoid life histories very different from any other insect group. The evolution of parasitoidism and the subsequent co-speciation of host and parasitoid species through time has contributed the majority of species diversity to the hyperdiverse lineage of Hymenoptera, with around 153,000 named species in total [5], and possibly up to ten times that number considering undescribed diversity [6]. Both the Chalcidoidea and Ichneumonoidea, considered by themselves, may comprise as many as 500,000 species each, representing one of the largest post-Cretaceous insect radiations [7–9]. In fact, the parasitoid Hymenoptera are a dominant force shaping the population dynamics of other arthropod species world-wide, providing essential ecosystem services via population regulation [6].

Parasitoid diversity may have been propelled by the evolution of a tremendous diversity in life histories. Strategies range from attacking hosts internally to externally, or from tricking the host into behaving normally post oviposition and during development to causing complete incapacitation of the host immediately [10, 11]. Besides the characteristic forms of insect-specific carnivory or “entomophagy”, some parasitoid Hymenoptera (Cynipoidea, Chalcidoidea, a few Braconidae, and a few sawflies) have diversified to use plant tissues as their hosts. This type of life history is known as plant galling, which is widespread also in many other arthropods, as well as nematodes.

Cynipoid wasps, the focus of this study, are a group that includes both a large diversity of gall-forming, as well as parasitoid lineages. This breadth of life histories presents a unique evolutionary conundrum for biologists studying cynipoid wasps: how does a hyper-specialized, gall-inducing phytophagous insect evolve from parasitoid (entomophagous) origins or vice versa? To add to the puzzle, some cynipoid lineages have adapted to usurp other species’ galls, a form of parasitism called inquilinism. Could inquillinism be a key step between gall induction and parasitoidism, as was suggested already by Malyshev [12]?

Phylogenetic research within cynipoid wasps has been pursuing these very questions for over 25 years now, based primarily on the ground-breaking work of Ronquist [13, 14]. While the majority of the earlier phylogenetic work has focused to a greater extent on the gall wasps, more recently, the figitids have received a considerable amount of attention in phylogenetic research [15–18]. Research on figitids began in earnest with Fontal-Cazalla et al. [16] focusing on eucoilines, a diverse subfamily of figitids parasitizing flies, and this led to larger analyses on figitids by Buffington et al. [17, 18]. Figitids have not been as easy to circumscribe as cynipids: both morphologically and biologically some lineages overlap in characters with cynipids, resulting in some taxa being classified in either family through time (e.g. *Euceroptres*) [19]. Considering these close affinities, obviously one cannot readily interpret the cynipid evolutionary tree without a comprehensive figitid phylogeny.

Through this series of influential phylogenetic studies, certain hypotheses for cynipoid evolution have been postulated: (1) a group of wood-boring wasps, the family Ibaliidae, are considered the earliest diverging lineage within cynipoids, suggesting the ancestors of all cynipoids possessed this biology; (2) the inquilinous gall wasps are close relatives of their host gall wasps (agastoparasitism); (3) the two more derived cynipoid families Cynipidae and Figitidae are sister-groups of each other [13–15, 20–22]. Ronquist et al. [20] summarizes these three core concepts, and also provided an updated tribal classification and identification key of the cynipids. While cynipid genera here are grouped at a tribal level, with all tribes belonging to one subfamily Cynipinae, groups of genera in the figitids are usually treated at the subfamily level, with tribes only present within the subfamily Eucoilinae. This is further summarized in Buffington et al. [23], which is the classification we apply throughout the present study.

Our present work is intended to thoroughly re-examine previous hypotheses on cynipoid evolution outlined above, by addressing some obvious deficiencies in previous studies involving cynipoid phylogenetics. First, previous studies lacked a comprehensive simultaneous analysis of *all* lineages of cynipoids. The need to remedy this has long been recognized by experts. A merged data set from several studies [17, 18, 21, 22, 24] helped in generating the latest phylogeny of Ronquist et al. [20], but the taxon sampling here was focused on Cynipidae. Many additional lineages have been better circumscribed and understood since the previous large-scale analyses (e.g. Thrasorinae, Pycnostigminae and Mikeiinae in the Figitidae; Diastrophini, Ceroptresini, Aulacideaini and Phanacidini in the Cynipidae), and their inclusion in a complete cynipoid matrix is certainly needed.

Second, the influence of outgroup choice on tree topology and ingroup relationships is well documented in phylogenetics [e.g., 25, 26, 27]. The placement of the superfamily within the larger Proctotrupomorpha (a clade also containing superfamilies Platygastroidea, Proctotrupoidea, Chalcidoidea and Diapriioidea) has generally been accepted, albeit no previous analyses of cynipoid relationships have included members of other Proctotrupomorpha as outgroups. Likewise, since larger Hymenoptera studies by Heraty et al. [28] and Sharkey [29], a platygastroid + cynipoid sister-group relationship had consistently been recovered, yet platygastroids have not been utilized as an outgroup for cynipoid phylogenetics. Instead, ibaliid wasps have consistently been used as an outgroup [13, 14, 17, 18, 20], resulting in a somewhat predetermined set of relationships among ingroup taxa.

Lastly, large-scale evolutionary studies are currently dramatically benefitting from modern genomic-based approaches, yet no attempts have been made to apply these methods to elucidate the evolution of cynipoids. These approaches include, for example, target enrichment of ultraconserved elements (UCEs) or anchored hybrid enrichment (AHE), both of which are extremely economical techniques for generating a wealth of genomic data from relatively small amounts of insect tissue [e.g., 30, 31–36]. These methods, paired with multiplexed sequencing, provide an obvious advantage in the sheer scale of data that can be generated and analyzed, data critical for resolving closely related taxa as well as estimating clade divergence. Just as important is the fact that high-quality UCE data can be generated from sub-par condition specimens [37–39], which revolutionizes taxon sampling strategies in general. Pinned specimens from museum collections (even several decade-old specimens) have become ideal candidates for UCE projects, resulting in more complete taxon sampling and eliminating complicated genomic preservation techniques during field work.

We present here the first comprehensive phylogenomic analysis of cynipoid relationships. The UCE approach was adopted for its demonstrated ability to utilize museum specimens [e.g., 37], allowing us to sample from all lineages of extant cynipoids housed in the United States National Museum of Natural History at Smithsonian Institution (USNM). A final matrix of 119 taxa from across the superfamily, as well as seven non-cynipoid outgroup taxa are included in the analyses (Additional file 1). Beyond a robust topological treatment in a maximum likelihood framework, we estimated a time-calibrated phylogeny using the most reliable published cynipoid fossils and reconstructed the evolution of parasitoidism, galling, and inquilinism, with a special focus on different host associations for gall makers. Given the nature of the dataset, both in scope and depth, it is not too surprising that our results are in many ways fundamentally different than all previous phylogenies published on this group. We discuss possible interpretations of these new phylogenetic results in the light of previous hypotheses, and also provide a discussion of potential evolutionary trajectories for the evolution of life history strategies in cynipoids.

## Results

### UCE data characteristics

The concentrations of our DNA extractions ranged from < 0.05 ng/µL to 27.4 ng/µL (average 2.9 ng/µL), due to variable specimen size and age range. Our post-library preparation DNA concentrations were equally variable, ranging from 0.1–151 ng/µL (average 31.6 ng/µL), with a total DNA input ranging from < 5.0–567 ng. From the enriched libraries, we were able to generate 219,391–21,713,730 raw sequencing reads per taxon (average: 3,134,133), which were assembled into 2,697–275,787 contigs (average: 51,162) with Trinity, of average length 254–394 bp per taxon (average: 314 bp). The assembled contigs matched 125–1,842 UCE loci (average: 1,058 loci) after removal of duplicates in PHYLUCE, with an average length of 235–708 bp per taxon (average across taxa: 389 bp). More details on the library concentrations and UCE capture values can be found in Additional file 2. We calculated several descriptive statistics from our alignments, which are listed in Additional file 3. We had filtered our alignments according to three different levels of taxon completeness (50%, 60% and 70%), meaning a locus had to be recovered in a certain percentage of the taxa to be retained. The 50%, 60% and 70% completeness matrices consisted of 1147, 918 or 626 loci, respectively, while having a total alignment length of 377,717 bp, 309,881 bp and 217,786 bp, respectively. Alignments had between 0.31–0.38 missing data, 0.72–0.74 variable sites, and 0.58–0.60 parsimony informative characters. GC content of all alignments was 0.43, whereas per taxon (i.e. sequence) GC content ranged from 0.40–0.46 among ingroup taxa and 0.40–0.49 among outgroup taxa (average 0.44; Additional file 2) across the aligned 50% completeness matrix. GC content across loci in the 50% completeness matrix ranged widely from 0.20–0.64, which led us to explore the influence of varying GC content of UCE loci on phylogenetic inference in more detail (Additional files 4, 5).

### Phylogeny of Cynipoidea

**Fig. 1:**
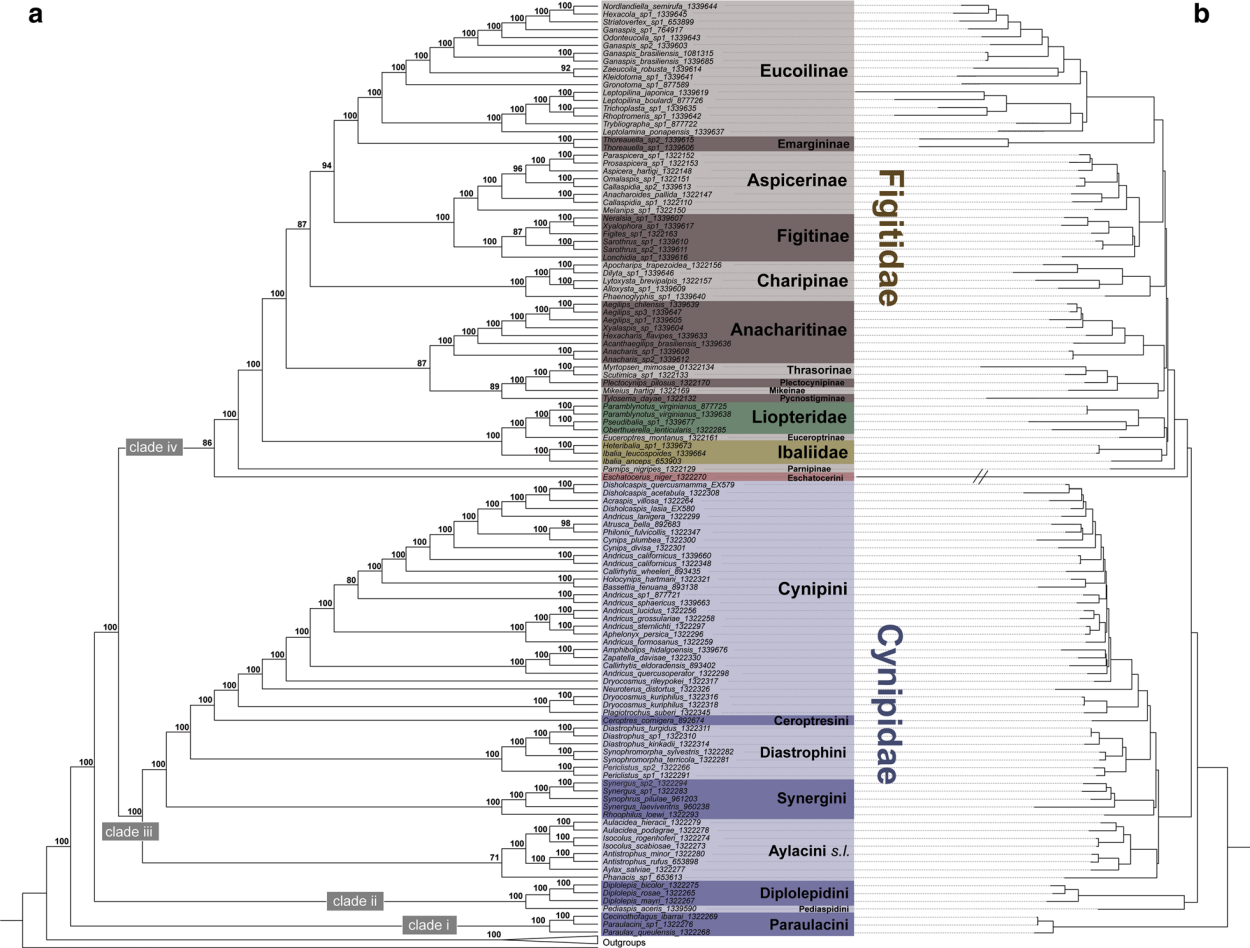
Phylogeny of Cynipoidea. Maximum Likelihood tree resulting from IQ-TREE analysis (combined ML search for best tree and 1000 bootstraps) of the 50% completeness matrix using SWSC-EN partitioning scheme. The analysis was rooted using the outer outgroup *Callihormius bifasciatus*. Main tree is displayed as cladogram for clarity of relationships (left panel); right panel shows phylogram including information on branch lengths. Bootstrap support values are depicted next to respective nodes. Current subfamily (for Figitidae) and tribe (for Cynipidae) assignments are indicated

Maximum likelihood analyses produce well-supported trees, with most nodes having bootstrap support (BS) = 100. Topologies resulting from analyses of the three taxon completeness matrices and their partitioned and unpartitioned variants are mostly in agreement with each other (Fig. 1 and Additional files 6, 7). As our main phylogenetic result, we therefore summarize major subfamily and tribal relationships recovered by the partitioned analysis of the 50% completeness matrix (ML-part-50; shown in Fig. 1), as this analysis received the highest bootstrap values across the phylogeny, while referring to the remaining completeness matrices and analyses only in case of deviations. Results are fully supported by BS = 100 unless reported otherwise.

I. Higher-level phylogeny of Cynipoidea and Cynipidae:
1. Cynipidae are recovered as not monophyletic, with the tribes Paraulacini, Pediaspidini, Diplolepidini, and Eschatocerini grouping outside of the family.
2. Our phylogeny supports four major lineages within Cynipoidea: **clade i**) containing the cynipid tribe Paraulacini, **clade ii**) containing the cynipid tribes Diplolepidini + Pediaspidini, **clade iii**) containing the cynipid tribes Aylacini s.l. (sensu lato, including Aulacideini and Phanacidini), Synergini, Diastrophini, Ceroptresini and Cynipini, and **clade iv**) containing the cynipid tribe Eschatocerini, the families Liopteridae and Ibaliidae, and all subfamilies of Figitidae.
3. The sister lineage to all other Cynipoidea are Paraulacini (= **clade i**), while Diplolepidini and Pediaspidini form a **clade ii**, which is sister to the remaining Cynipoidea excluding Paraulacini.
4. The remaining cynipid tribes (Aylacini s.l., Synergini, Diastrophini, Ceroptresini, Cynipini) together form **clade iii**, with each individual tribe recovered as monophyletic. In the following, we will refer to this clade iii as Cynipidae s.s. (sensu stricto).
II. Phylogeny of Figitidae s.l.:
5. Cynipidae s.s. are the sister group to **clade iv** comprising the cynipid tribe Eschatocerini, the families Liopteridae and Ibaliidae, and all Figitidae. We refer to this clade as Figitidae s.l.
6. Within **clade iv**, Eschatocerini (currently classified in Cynipidae) has moderate support as sister to all remaining taxa (BS = 86, Fig. 1), followed by full support for the figitid subfamily Parnipinae as sister to remaining members.
7. A clade consisting of Ibaliidae + (Liopteridae + figitid subfamily Euceroptrinae) is further sister group to all remaining figitid lineages within clade iv. Ibaliidae and Liopteridae are recovered as monophyletic by our data.
8. We refer to the clade including all remaining figitid lineages (excluding Eschatocerini, Parnipinae, Euceroptrinae, Ibaliidae and Liopteridae) as Figitidae s.s.. All subfamilies of Figitidae s.s. with multiple representatives included (Eucoilinae, Aspicerinae, Figitinae, Charipinae, Anacharitinae and Thrasorinae) are recovered as monophyletic.
9. Within Figitidae s.s., we recover a clade consisting of the smaller subfamilies Thrasorinae, Plectocynipinae, Pycnostigminae and Mikeinae, which together form the sister group of Anacharitinae, albeit with only moderate support (BS = 87, Fig. 1).
10. Eucoilinae and Emargininae further are sister lineages, in a larger clade with sister group Aspicerinae + Figitinae with good support (BS = 94, Fig. 1). Charipinae are moderately supported (BS = 87, Fig. 1) as sister to this clade consisting of the former four subfamilies.

Within the Figitidae s.s. and Cynipidae s.s., we see some rearrangements of relationships between subfamilies or tribes derived from the remaining matrices and partitioning schemes, mainly in areas where support was not 100% in the preferred topology (ML-part-50). Within Figitidae s.s. in the partitioned and unpartitioned analysis of the 70% completeness matrix (ML-part-70, Additional file 6; ML-unpart-70, Additional file 7), Charipinae are sister to Aspicerinae + Figitinae only, instead of a larger clade that also includes Eucoilinae + Emarginae as in the remaining analyses. Further, in the ML-unpart-70 tree (Additional file 7) the clade consisting of Thrasorinae, Plectocynipinae, Mikeinae and Pycnostigminae is recovered as sister to Eucolinae + Emarginae (not as sister to Anacharitinae as in the remaining analyses), but support for this alternative result is low (BS = 65). In general, support values for higher-level relationships within Figitidae s.s. are reduced within the results from the ML-part-60 and ML-part-70 (Additional file 6), as well as ML-unpart-50, ML-unpart-60 and ML-unpart-70 (Additional file 7) data sets, compared to our preferred ML-part-50 topology (Fig. 1).

Within Cynipidae s.s., only one taxon is moving between analyses: *Phanacis*. In the ML-part-50 and ML-part-70 trees (Fig. 1 and Additional file 6B), *Phanacis* is part of Aylacini s.l. with moderate bootstrap support (BS = 71–75), whereas the taxon is sister to the remainder of Cynipidae s.s. in the ML-part-60 (Additional file 6A), ML-unpart-60 (Additional file 7B), and ML-unpart-70 (Additional file 7C) trees with full support (BS = 100). In the ML-unpart-50 tree, *Phanacis* is placed as sister to the clade consisting of Cynipini, Ceroptresini, Diastrophini, and Synergini, but this grouping is basically unsupported (BS = 45). Thus, it remains unclear whether *Phanacis* is a member of, or just a close relative of Aylacini s.l.. The relationships between members of this clade otherwise remain stable.

The coalescent tree estimated with ASTRAL-III v5.6.3 from the 1143 gene trees is overall not as well supported as the concatenated analyses, with many branches having local posterior probabilities (LPP) < 0.9 (Additional file 8). All four main clades as outlined above are recovered; however, relationships especially within clade iv change significantly, although with low support in most cases. Specifically, the clade Figitidae s.s. in this analysis is not recovered as monophyletic, but broken up into two clades by a clade containing Ibaliidae, Euceroptrinae and Liopteridae. One clade hereby contains Eucolinae and Emarginae and another clade contains the rest of the figitid subfamilies (but receives poor support, LPP = 0.43, Additional file 8). Parnipinae and Eschatocerini are the earliest branching lineages of clade iv, similar to results from the concatenated analyses. However, Eschatocerini are poorly supported in this position (LPP = 0.6, Additional file 8). Relationships within the Cynipidae s.s. clade are similar to those recovered by the ML-part-50 and in the ML-part-70 tree with *Phanacis* as sister to the rest of Aylacini s.l.. Paraulacini (clade i) are recovered as sister to all of Cynipoidea in the coalescent tree as well. However, the clade Diplolepidini + Pediaspidini (clade ii) is here sister to Cynipidae s.s. (not to rest of Cynipoidea excluding Paraulacini).

### Sensitivity analyses

#### Rooting

Since the placement of families Ibaliidae and Liopteridae was within Cynipoidea in all our analyses (which presents a significant difference to most previous results) we tested whether this was a result of (incorrect) outgroup rooting in previous analyses or could be novel evidence provided by our UCE data. We excluded all non-cynipoid outgroups from the analyses and placed the root on the ibaliid branch [similar to 20]. The higher-level relationships resulting from these analyses are summarized in Additional file 9 (panel A, but see Additional file 10 for full cladograms) and show several major differences to the results from the analyses rooted with the correct non-cynipoid outgroups. When rooting on the ibaliid branch, Liopteridae and *Euceroptres* are pulled out of Figitidae s.l. and group as sister to Cynipoidea (excl. Ibaliidae). Clade i and ii (Paraulacini and Diplolepidini/Pediaspidini), which were recovered as the earliest branching lineages in almost all our analyses, are now nested within Cynipoidea as sister-group to Cynipidae s.s., with Parnipinae and Eschatocerini grouping as sister to the former combined. While these results are not directly comparable with Ronquist et al. [20] tree, which was much less resolved than ours, they are much more similar to that result [20; depicted for comparison in Additional file 9B] than to our topology estimated using the correct outgroup rooting. The SH test did not detect a significantly better likelihood score for the tree estimated by rooting with *Paraulax* versus rooting with *Ibalia* versus specifying no outgroup at all. We surmise that the novel positions of Paraulacini and Diplolepidini/Pediaspidini as earliest branching taxa, and Ibaliidae and Liopteridae as derived members of Cynipoidea, are indeed the result of proper outgroup taxon sampling and correct rooting in our analyses.

#### Position of *Eschatocerini*

An interesting, seemingly considerably derived taxon in our data set are the monotypic Eschatocerini, represented by *Eschatocerus niger*. All of our variations of ML analyses, as well as coalescent analyses place *E. niger* as sister group to the rest of clade iv (Figitidae s.l.), albeit sometimes with lower support, and this taxon always possesses a distinctly long branch. We first explored whether *E. niger* had influence on the position of other taxa in the analyses in the sense of a “rogue” taxon, by simply performing ML analyses excluding this taxon. Results from these exclusion analyses proved identical to the results from analyses with the same settings while including *E. niger* (compare Additional file 11A with Additional file 7A, and Additional file 11B with Additional file 7C), and thus did not indicate any “rogue” behavior of this taxon.

Secondly, we investigated whether the position of *E. niger* could be the result of particular characteristics of this data set and its locus and taxon composition. As noted above, the GC content across all taxa in our data set did not vary greatly (0.40–0.46 among ingroup taxa), and GC content for *E. niger* was on the lower end of this spectrum (0.41, Additional file 2). However, GC content across loci varied significantly (0.20–0.64), which piqued our curiosity as to how this affected phylogenetic inference, in general and with respect to *E. niger*. We investigated this variation by binning our UCE loci depending on their GC content, and performed phylogenetic inference (ML-IQTREE analyses) on a concatenated matrix from each bin. The results of this binning experiment are summarized in Additional file 4 and Additional file 12. Higher-level relationships of Cynipoidea did not change in results from bins compared to our main topology and its alternatives (see above)—with the exception of the position of *E. niger*. The binned analyses identified three scenarios for the placement of this taxon (Additional file 12): A—*E. niger* is sister to rest of Figitidae s.l., as estimated in all our unbinned analyses, B—*E. niger* is sister to Cynipidae s.s., and C—*E. niger* is sister to all Cynipoidea excluding Paraulacini. GC content did not correlate with the recovered position of *E. niger*. The summary of results in Additional file 4 shows that the position of *E. niger* changes with changing GC content, but without a clear trend. Position C is only recovered by two bins with low GC content (0.37–0.41), but results from the lowest GC content recover *E. niger* again in position A (0.26–0.37). Position B is most prevalent in bins with high (0.51–0.64) and medium GC content (0.44–0.47), whereas position A is recovered from a variety of GC contents. In most cases, any position of this taxon is relatively well supported, with the exception of trees from bins 2 (BS = 51, Additional file 4) and 5 (BS = 70, Additional file 4). In summary, although GC content appears to some degree correlated with the phylogenetic placement of *E. niger*, the results from this comparison remain inconclusive. It is possible that other intrinsic characteristics of the UCE loci in our data set are responsible for the observed patterns, which are imperfectly (or not at all) correlated with GC content.

### Timescale of the evolution of Cynipoidea

**Fig. 2:**
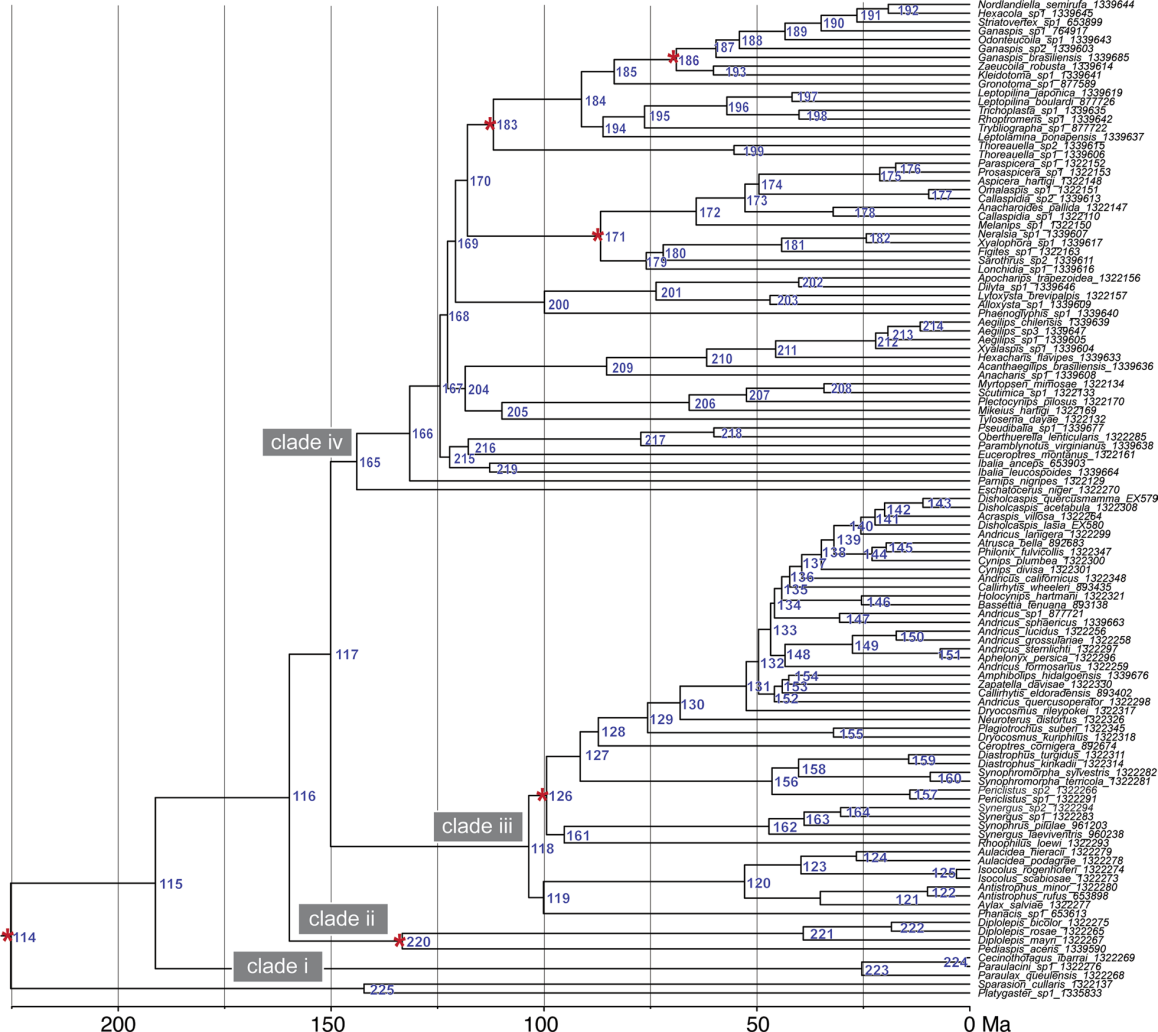
Timescale of cynipoid evolution. Time-calibrated phylogeny of Cynipoidea from dating analyses using approximate likelihood in mcmctree and codeml as part of the PAMLv4.9 package. Displayed are results estimated using the median age range (211–236 Ma) calibration on the root (see main text for details) and five fossils within the Figitidae and Cynipidae. Placements of fossil calibrations are indicated by a red star on the respective nodes; Additional file 5 specifies fossil information. Blue node numbers refer to Additional file 13 which summarizes divergence estimates across all calibration schemes

We estimated divergence times for Cynipoidea using five fossil calibrations and three secondary age range estimates to calibrate the root node (Additional file 5). Figure 2 presents a time-calibrated phylogeny for the analysis using the median age range on the root; a summary of divergence ages for major clades, tribes and subfamilies across our three different root age calibration is given in Table 1, and a comprehensive summary of results for all analyses can be found in Additional file 13. In the following, we refer for brevity to median ages in the text only, but 95% HPD (highest posterior density) intervals can be found in Table 1.

**Table 1:** Crown group age estimates for major clades within Cynipoidea

Clade	Node	Median root range			Maximum root range			Minimum root range		
		Median	Min	Max	Median	Min	Max	Median	Min	Max
Cynipoidea	115	191.2	151.4	230.8	195.0	149.2	245.9	190.5	148.7	234.3
Clade I: Paraulacini	223	25.3	7.9	48.3	24.9	8.2	46.4	24.8	8.2	46.1
Clade ii: Diplolepidini + Pediaspidini	220*	141.9	96.0	192.2	135.2	104.3	172.0	133.0	100.7	167.6
Diplolepidini	221	39.1	17.8	63.5	38.9	17.8	62.9	38.6	17.9	62.2
Split clade ii—rest of Cynipoidea	116	159.8	124.0	199.3	160.2	124.6	201.6	159.1	123.8	195.6
Split clade iii—clade iv	117	150.1	115.9	188.8	150.3	116.5	190.3	149.5	115.7	184.5
Clade iii: Cynipidae s.s.	118	103.5	71.6	141.7	97.1	71.9	135.3	101.0	72.8	136.3
Aylacini s.l. (incl. *Phanacis*)	119	100.1	68.6	137.8	93.8	68.6	131.5	97.7	69.9	132.6
Aylacini s.l. (excl. *Phanacis*)	120	52.9	30.8	76.5	51.6	31.8	72.4	52.1	31.8	73.5
Synergini	161	95.2	64.7	132.6	89.0	64.0	126.0	92.9	65.5	128.1
Diastrophini	156	46.4	25.4	69.0	45.0	26.1	66.2	45.5	26.4	66.9
Cynipini	129	75.6	50.5	106.9	71.2	50.8	97.6	73.8	51.9	102.0
Clade iv—Figitidae s.l.	165	143.9	111.3	181.5	144.3	112.0	182.5	143.5	110.9	177.2
Split Parnipinae—Ibaliidae/Liopteridae/Figitidae s.s.	166	131.5	102.8	166.0	132.5	103.1	166.2	131.3	102.6	162.4
Split Ibaliidae + Liopteridae/ Euceroptrinae—Figitidae *s.s*	167	124.4	98.0	157.6	125.8	98.1	157.1	124.3	97.3	154.4
Ibaliidae + Liopteridae/Euceroptrinae	215	122.1	95.6	154.8	123.6	96.1	154.6	122.1	95.0	151.9
Ibaliidae	219	112.7	80.3	148.9	114.3	80.2	149.3	112.8	80.7	147.4
Liopteridae	217	77.2	46.4	110.4	78.4	48.6	107.5	77.1	47.1	106.9
Figitidae s.s	168	122.6	96.6	155.4	124.1	96.9	155.0	122.6	96.0	152.4
Pycnostigminae (Mikeinae (Thrasorinae, Plectocynipinae)	205	109.8	79.3	141.7	111.4	80.9	143.2	109.9	79.1	140.5
Anacharitinae	209	85.3	55.2	117.7	85.7	57.8	115.6	84.9	56.5	115.1
Charipinae	200	99.9	68.7	135.0	101.0	70.2	135.3	99.8	69.1	132.1
Figitinae	179	75.9	55.4	97.7	77.4	56.4	98.6	76.2	54.9	97.6
Aspicerinae	172	64.2	43.7	84.5	64.7	44.9	84.8	64.0	44.3	83.7
Emargininae	199	55.3	29.4	82.3	56.0	29.4	83.5	54.9	29.3	82.3
Eucoilinae	184	91.1	69.8	117.6	91.0	72.1	114.5	90.4	71.0	113.8

Presented are median ages and 95% HPD intervals across three separate sets of MCMCTREE analyses, implementing different root calibrations. Crown group ages are given for major lineages. Node numbers refer to Fig. 2. For a full summary of results from all analyses, refer to Additional file 13. For calibrated nodes (indicated by *), median root range estimates are given from analyses that excluded this calibration

Median crown age estimates for Cynipoidea center around 190 Ma (Fig. 2 and Table 1: node 115), suggesting the origin of the superfamily in the early Jurassic period. Crown-group Paraulacini are estimated quite young with an Oligocene age of ca. 25 Ma (Fig. 2 and Table 1: node 223), indicating that this tribe may have seen large amounts of extinctions since its divergence from the remainder of Cynipoidea. Clade ii, including Diplolepidini and Pediaspidini, is estimated with an age of ca. 142 Ma (Fig. 2 and Table 1: node 220) to have originated in the early Cretaceous; in contrast, crown-group Diplolepidini (the rose gallers) have an Eocene origin and are only about 39 Ma old (Fig. 2 and Table 1: node 221).

Cynipidae s.s. (clade iii) are estimated with a late Cretaceous origin around 97–104 Ma (Fig. 2 and Table 1: node 118), with diversification at the tribal level taking place between ~ 45 and 100 Ma. Diastrophini are estimated as the youngest tribe with Cynipidae s.s., with a crown age of 45–46 Ma (Fig. 2 and Table 1: node 156) in the Eocene. The oak-galling Cynipini are estimated with an origin between 71–76 Ma in the late Cretaceous (Fig. 2 and Table 1: node 129), while Synergini are somewhat older with an estimated age of ca. 89–95 Ma (Fig. 2 and Table 1: node 161) within the same geological time period.

Similar to clade ii, crown Figitidae s.l. (clade iv) are estimated to be of an early Cretaceous origin ca. 143–144 Ma (Fig. 2 and Table 1: node 165), equivalent with the divergence from Eschatocerini. Subsequent higher-level diversification within this clade happened fairly fast within the next 10–25 million years within the early Cretaceous. The subfamily Parnipinae diverged about 10 Ma later, ca. 131–132 Ma (Fig. 2 and Table 1: node 166), followed shortly afterwards by the Ibaliidae/Liopteridae/Euceroptrinae clade ca. 124–126 Ma (Fig. 2 and Table 1: node 167). Within this latter clade, Ibaliids are much older than Liopteridae, with an estimated age of 113–114 Ma (early Cretaceous) vs 77–78 Ma (late Cretaceous), respectively (Fig. 2 and Table 1: nodes 219 vs 217). Crown Figitidae s.s. are estimated to be around 123–124 Ma old (Fig. 2 and Table 1: node 168) with an early Cretaceous origin. The lineage composed of Pycnostigminae, Mikeinae, Thrasorinae and Plectocynipinae is the oldest within this clade with an estimated early Cretaceous age of 110–111 Ma (Fig. 2 and Table 1: node 205). The other subfamilies within Figitidae s.s. originated throughout the late Cretaceous until the Paleocene (101–55 Ma), with Charipinae hereby estimated as oldest (100–101 Ma; Fig. 2 and Table 1: node 200) and Emargininae as the youngest lineage (55–56 Ma; Fig. 2 and Table 1: node 199).

### Evolution of life histories

#### Results from three-state model

**Fig. 3:**
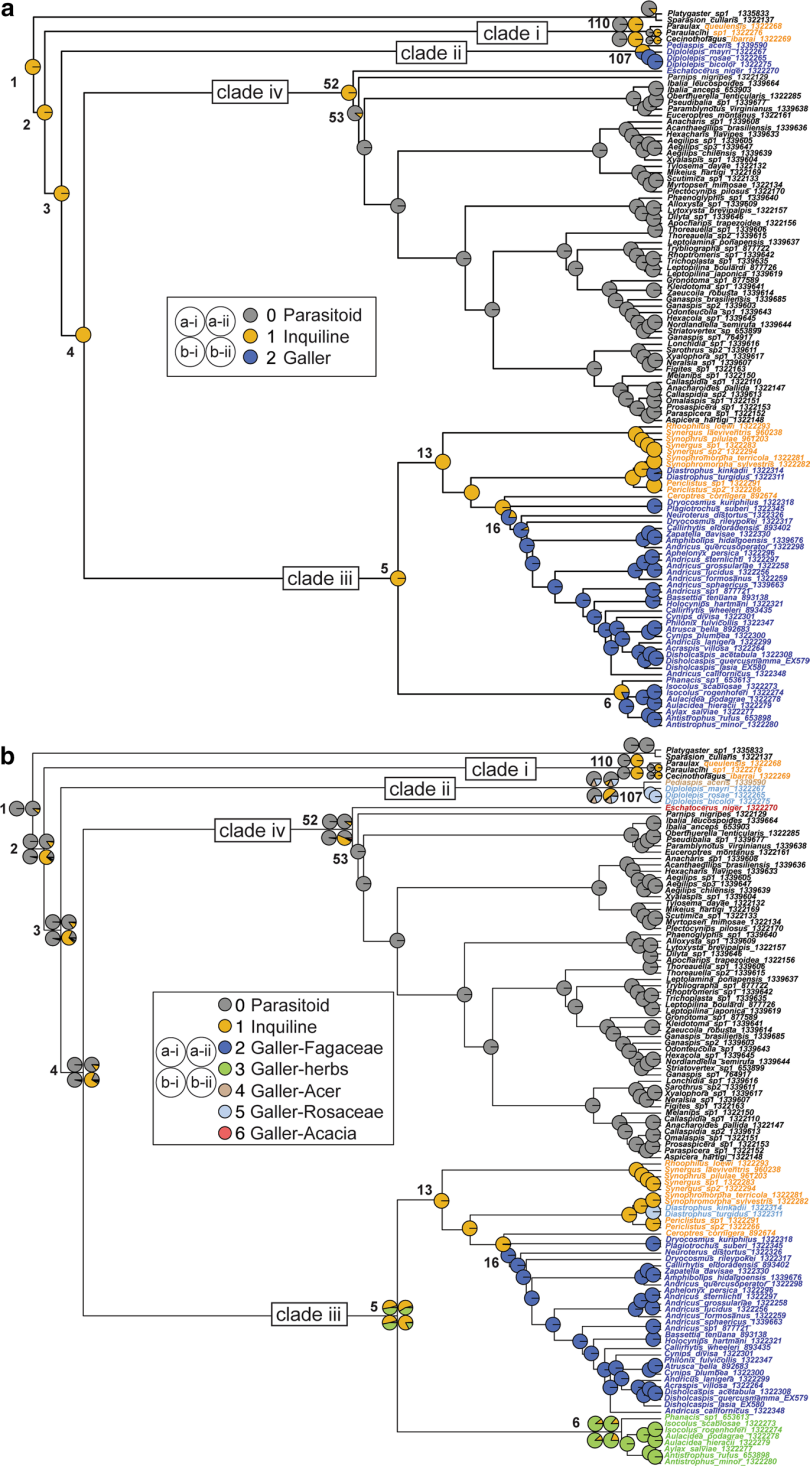
Evolution of cynipoid life histories. Ancestral state reconstructions for cynipoid life histories using maximum likelihood and the rayDISC function in the R package corHMM. Summarized are exclusively results from four analyses for which uncertain taxa were coded as parasitoids. **a** 3-state set, with states 0/grey = parasitoid, 1/orange = inquiline and 2/blue = galler. **b** 7-state set, with states 0/grey = parasitoid, 1/orange = inquiline and 2/dark-blue = galler-Fagaceae, 3/green = galler-herbs, 4/light-brown = galler-*Acer*, 5/light-blue = galler-Rosaceae, 6/red = galler-*Acacia*. Each panel represents a summary of four separate variant reconstructions for each of the two trait sets. Only reconstructions of the best fitting models are shown, which were the ARD-model for the three-state set and the ER-model for the seven-state set. Pie proportions represent state probabilities estimated for each internal node. We present all four variant reconstructions only for nodes were estimations differed between these analyses; **a-i** = Outgroups included, Paraulacini are coded as parasitoids; **a-ii** = Outgroups included, Paraulacini are coded as inquilines; **b-i** = Outgroups excluded, Paraulacini are coded as parasitoids; **b-ii** = Outgroups excluded, Paraulacini are coded as inquilines. Taxon labels are colored by their terminal’s state; select node are labeled with numbers corresponding to Additional file 14 and 15

We performed ancestral state reconstructions for several variations of two main life history data sets for cynipoids. For the three-state reconstructions, the ARD model was chosen as best fitting by the LHT test, whereas for the seven-state reconstructions the ER model was chosen as the best fitting model. Results summarized in Fig. 3 show only results for the best-fitting model. Virtually no differences in results were observed between coding the ten “assumed parasitoids” as parasitoids vs coding these taxa as unknown; for simplicity we therefore display only results where these were coded as parasitoids. A full summary of estimated states can be found for each node in Additional files 14 and 15. Members of Paraulacini are either inquilines or parasitoids within chalcidoid galls on *Nothofagus* in southern Chile [40]. As expected, including vs excluding the outgroups and coding Paraulacini (i.e. clade i in Fig. 3) as parasitoids vs inquilines had an effect on the reconstruction of the ancestral lifestyle of Cynipoidea, but only within the seven-state model. In the three-state model, the ancestral Cynipoid is estimated to be an inquiline (Fig. 3a, node 2) with probability = 1.00 (Additional file 14) in all reconstructions. Unsurprisingly, the ancestral state estimated for the most recent common ancestor (MRCA) of clade i follows closely the assigned states for the terminal taxa within Paraulacini, and is consequently estimated to be either a parasitoid or an inquiline.

For the remainder of the reconstructions all variations of the three-state model agree on the most probable state and show only slight variations in probability percentages (Fig. 3a and Additional file 14). Inquilinism is the dominant lifestyle throughout the early evolution of cynipoids in the three-state model, with clade ii, iii and iv having a common inquiline ancestor (P = 1.00, node 3; Fig. 3a and Additional file 14). This lifestyle is retained in the MRCA of Figitidae s.l. (clade iv) until a transition to a parasitoid lifestyle at node 53 for all Figitidae s.l. (excluding *E. niger*). While gall-making is the dominant behavior in Cynipidae s.s. (clade iii), members of this clade ancestrally appear to have been inquilines and gall-making is the more derived state, evolving once in Diastrophini (*Diastrophus*)*,* once in Cynipini, and once in Aylacini s.l.. The MRCA of the latter tribe is still estimated to be an inquiline with high probability (0.82–0.84; node 6, Fig. 3a and Additional file 14). Overall, the three-state model does not support the hypothesis that parasitoidism was retained from a common ancestor with Platygastroidea, but suggests one or two origins of parasitoidism later in the evolution of Cynipoidea. Instead, inquilinism has the full support as ancestral cynipoid lifestyle, a behavior which is relatively rare in extant cynipoids.

#### Results from seven-state model

Reconstructions within the seven-state model provide a completely different view on the early evolution of cynipoid life histories (Fig. 3b). Overall, dividing gallers into separate categories according to their host plant may have resulted in excessive weighting of the parasitoid state. Parasitoidism is estimated to be dominant throughout the early evolution of cynipoids (node 2–4, Fig. 3b) in the seven-state model. The MRCA of Cynipoidea is a parasitoid with high probability (P = 0.85–1.00, node 2, Fig. 3b and Additional file 15) in most reconstructions, with the exception of the analysis that excluded outgroups and coded Paraulacini as inquilines. In this case, inquilinism receives highest support at nodes 2–4 (P = 0.73–78, Additional file 15). For clade I (Paraulacini), estimations are unchanged compared to the three-state model, with estimates either suggesting a parasitoid or an inquiline MRCA with highest probability (node 110, Fig. 3b and Additional file 15) depending on the terminal states. Clade ii contains both the Rosaceae-galling Diplolepidini and the *Acer*-galling Pediaspidini, and when these galling behaviors are treated as separate states, this clade is estimated with a parasitoid ancestor in most analyses (P = 0.57–0.67, node 107; Additional file 15)—only when outgroups are excluded and Paraulacini coded as inquilines, an inquiline ancestor becomes equally probable (P = 0.40–0.50, node 107; Fig. 3B and Additional file 15). In the seven-state model, a parasitoid MRCA is also most strongly suggested for the entire clade iv (Figitidae s.l.; node 52; Fig. 3B), but with arbitrary support when excluding outgroups and enforcing inquilinism in paraulacines (b-ii, Additional file 15). Reconstructions for clade iii resemble those of the three-state model more closely, including estimation of an inquiline ancestor for that clade with equivocal to moderate probability (P = 0.49–0.84, node 5, Fig. 3b), with the best alternative being a herb-galling ancestor. The herb-galling habit evolved only once and is most likely already present in the MRCA of all Aylacini s.l. (P = 0.78–0.88, Node 6, Fig. 3b and Additional file 15). Inquilinism is estimated as the likeliest ancestral state for the remaining Cynipidae s.s. (P = 1.00, node 13, Fig. 3b and Additional file 15), with a secondary transition to rose-galling (Rosaceae) in Diastrophini, and a single transition to oak-galling (Fagaceae) in Cynipini (P = 0.99, node 16; Fig. 3b and Additional file 15).

## Discussion

Our phylogenomic dataset, while supporting many previously published hypotheses, yields a number of unexpected results. It appears the inclusion of a platygastroid outgroup had a profound impact on the position of several ingroup clades, and thus on our understanding of cynipoid evolution. The Paraulacini emerged as a key group, a small group of gall wasps found on *Nothofagus* trees in the southern hemisphere. This taxon was found to be sister-group to the rest of Cynipoidea, suggesting either inquilinism or parasitoidism is the ancestral biological state of cynipoid wasps (depending on the character state model used). Consequently, Cynipidae was found to be not monophyletic. Perhaps more surprising is the recovery of Ibaliidae nested far within Cynipoidea and sister-group to Figitidae, resulting in an alternative hypothesis of cynipoid evolution. We discuss these and other new insights in depth in the following.

### Novel datasets yield unexpected topologies

There are three ‘firsts’ in our dataset that may have contributed to the novel relationships recovered: proper outgroup sampling, a genome-scale data set, and comprehensive ingroup sampling. This analysis is the first of its kind to use Platygastroidea as the outgroup for ingroup cynipoids. The typically used outgroup has been *Ibalia*, itself a cynipoid. Several treatments on cynipoid evolution rely on the early-branching position of *Ibalia* to articulate further hypotheses within Cynipoidea [13–15, 17, 18, 20]. The morphology of *Ibalia* has been suggested to be archaic [41]. The taxon indeed has many seemingly plesiomorphic states, most (if not all) associated with the parasitism of wood boring insect larvae.

Using platygastroids as outgroups, we found *Ibalia* much more derived than previously hypothesized, and instead Paraulacini was inferred as sister-group to the remaining cynipoids. These novel relationships were robust and well-supported in all our analyses and with respect to different analytical parameters. If we remove the platygastroid and other outgroups from the analysis and root with *Ibalia* (Additional file 9A), we return to a topology that nearly resembles those of previous family-level analyses [Additional file 9B,C; see 17, 18, 20]. It appears the position of *Ibalia* and the liopterids as sister-group to the Figitidae is the result of more comprehensive taxon and outgroup sampling, and not so much improved or novel information content within the UCE data itself. This more nested position of ibaliids has been observed before in larger-scale analyses of Hymenoptera by Heraty et al. [28], Sharkey et al. [42] and Peters et al. [43], where in all three cases *Ibalia* is found sister-group to Figitidae, and not sister-group to all cynipoids.

Have phylogenetic studies of cynipoids to this point been misled by previous interpretations of the morphology and biology of *Ibalia* as being ancestral? It appears so. We are probably missing fossil intermediates that may help interpret the new topology presented here. Perhaps the very nature of the biology of *Ibalia*, with its switch to being a wood-boring insect parasitoid, has shaped its morphology and thus resulted in an ‘archaic-looking’ wasp, morphologically more akin to other wood-boring hymenopterans.

### Paraulacini—the earliest diverging cynipoid?

Perhaps the most surprising result of this research is the recovery of the Paraulacini as the sister-group to all extant cynipoids. This taxon had been not included in most previous treatments [15, 18]. Ronquist et al. [20] did include Paraulacini in their analysis, however, and their total-evidence topology [Fig. 2 in 20] already indicated Paraulacini are certainly outside the Cynipidae s.s..

Our newly recovered topology with Paraulacini being sister to all remaining cynipoids (Fig. 1) raises some curious considerations for cynipoid evolution. Members of Paraulacini are either inquilines or parasitoids within galls on *Nothofagus* in southern Chile [40]. This is, in fact, the only known cynipid lineage indigenous to that region. The geographic distribution of Paraulacini, and their host plant, *Nothofagus*, suggests early origins of the taxon [44, 45]; this latter observation is supported by the divergence estimate of the split of this lineage from the remaining cynipoids around 175 Ma (Table 1). Sauquet et al. [46] suggested the crown age of *Nothofagus* to be between 13 and 113 MY old—a wide time bracket due to high sensitivity to calibration points [46], but consistent with the crown-group age of Paraulacini at 25 Ma (Table 1). The fossil record indicates that stem-group *Nothofagus* has been a major component of Gondwanan habitats since the Late Cretaceous [47] so it is possible that Paraulacini has been associated with *Nothofagus* for quite some time. Alternatively, early stem-paraulacines may have colonized a distant relative of *Nothofagus*, perhaps among the other Fagales. The inquilinous, or perhaps parasitoid nature of Paraulacini suggests the group represents a transition to a life history associated with galling systems, either as guest herbivores or entomophages of gall inducing Hymenoptera, but not gall inducers.

### Inquiline or parasitoid—first hypotheses of cynipoid evolution

The favored hypothesis supported by our three-state ancestral reconstructions is an inquiline biology dominating the early origin of cynipoids (Fig. 3a), in an “inquiline-first” scenario. If the biology of inquilinism was the ‘ground-plan’ life history strategy (a hypothesis that was originally proposed by Malyshev [12] for what we now refer to as apocritan Hymenoptera), this could better explain the evolution of inquilinism across the remaining cynipoid tree. This is the first glimpse of an entirely new interpretation of cynipoid evolution, that cynipoids are derived from gall-associated inquiline ancestors. Considering this proposed ancestral biology of Cynipoidea with respect to their closest relatives, the platygastroids, is especially intriguing. While scelionids are egg parasitoids of various arthropods ranging from grasshoppers, true bugs, and spiders, the platygastrids are parasitoids of gall-inducing insects, especially the dipterous Cecidomyiidae [48]. This inquiline-first scenario also has a possibility under the seven-state model, but is recovered only when Paraulacini are considered inquilines (and not parasitoids) and outgroups are excluded. Nieves-Aldrey et al. [40] argued convincingly that *Paraulax* and *Cecinothofagus* (i.e. Paraulacini) are either parasitoids of chalcidoid gall inducers, or inquilines of the same. Their phylogenetic position suggests that the early evolution of cynipoids was entomophagous in nature, while already being associated with galls.

Our more complex, seven-state ancestral reconstructions involving host plant data along with the galling behavior (Fig. 3b) showed parasitoidism as the most likely ancestral life history of the MRCA of Cynipoidea (followed by inquilinism, if Paraulacini are coded as inquilines) and throughout the early evolution of the superfamily. This “parasitoid-first" scenario, we could argue, presents an intuitively more logical progression of the evolution of host use in the cynipoids – if we can interpret inquilinism as an intermediate physiological trait somewhere between entomophagy (parasitoidism) and phytophagy (gall induction). The validity of this argument relies heavily on the distinction of a “simple” inquiline as opposed to a “lethal” inquiline. In the former, the inquiline is phytophagous, unable to make a gall itself, and perhaps drawing resources from the inducer; in the latter, not only does the inquiline need another wasp to make the gall, but the inquiline also kills the inducing wasp, possibly even consuming the host remains. Lethal inquilinism was suggested for the genera *Paraulax* and *Cecinothofagus* in Chile on *Nothofagus* trees by Nieves-Aldrey et al. [40]. Focused fieldwork on the *Nothofagus* system seems critical to answer the remaining questions on the life history of Paraulacini, but studying insects inside a closed system such as a gall is no trivial task, especially in the remote regions where *Nothofagus* trees occur. If these taxa are relicts of these early transitions in cynipoid evolution, studying their life history could provide some insight as to how this physiological transition between entomophagy and phytophagy could have come about.

In summary, the ancestral life history of cynipoids cannot be definitively resolved by our study, yet our results from employing a simple three-state model provide strongest support for an inquiline-first hypothesis. The seven-state model supports parasitoidism as the most likely ancestral state, but this result may be an artifact of galling being broken up into several states compared to parasitoidism represented as a single state.

### Relationships among Cynipidae

The Cynipidae were never recovered as monophyletic in any of our analyses using Platygastroidea as the outgroup (Fig. 1, Additional files 6, 7, 8, 11). Four tribes render Cynipidae paraphyletic: Eschatocerini, Paraulacini, Pediaspidini and Diplolepidini. We refer to the remaining Cynipidae as Cynipidae s.s. or clade iii (Fig. 1; Table 1.). These relationships are compatible with Ronquist et al. [20], but our data present much-needed improved resolution along the backbone of the tree. All four of the tribes mentioned above were found to be sister-group to the core cynipids in Ronquist et al. [20; Additional file 9B], with the node subtending Cynipidae s.s. receiving relatively low support.

The weakest relationships across our main phylogeny recovered (Fig. 1) are found within the herb gallers, Aylacini s.l.. This group was not monophyletic in Ronquist et al. [20], but the backbone of that tree was unresolved. In our analyses, all the herb gallers, excluding Phanacidini, were found monophyletic with BS = 100. Aylacini and Aulacideini were intermingled in our tree. However, *Aylax* was the only Aylacini we were able to include in the analysis here, and adding more members of this tribe may help resolve whether these two tribes are reciprocally monophyletic. Either way, we provide evidence here that herb galling, as a lifestyle, arose once within Cynipidae s.s. (discussed further below). The divergence of the herb gallers from the synergine inquilines (Synergini), the *Rubus* gallers (Diastrophini) and oak gallers (Cynipini) is estimated to have occurred at around 100 Ma. As *Rubus* is estimated to diverge from the rest of Rosaceae at around 78 Ma [49], our node age corresponds well with this estimate.

Among the three other Cynipidae s.s. tribes in which inquilinism occurs (Synergini, Diastrophini and Ceroptresini), the exclusively inquilinous Synergini [sensu 20] were recovered monophyletic in our analysis. We see strong support for the agastoparasitism hypothesis [where host and parasite are close relatives of each other, suggesting there are shared physiological traits among the two species; 13] regarding *Synophromorpha*, *Periclistus* and *Diastrophus*, where *Diastrophus* are gall-inducers, and the other two genera inquilines of these inducers. The Ceroptresini are only represented by a single species here, but the placement of this taxon as sister-group to Cynipini is well supported.

Unsurprisingly, our data recovered a strongly supported monophyletic Cynipini. We recover a crown-group age of ca. 80 Ma for Cynipini, while Sauquet et al. [46] recovered a crown age of 85 Ma for Fagaceae. Cynipini appear to have diversified very rapidly following the proliferation of *Quercus*. Hipp et al. [50] posited that the subgenera of *Quercus* diverged around 50 Ma, and our divergence analyses indicate that this is when major generic diversification within Cynipini happened as well. The rapid diversification within Cynipini is also evidenced by the very short internal branches estimated in the maximum likelihood analyses (Fig. 1). Nearly all cynipine ‘genera’ sampled here were found to be not monophyletic in our analyses, and clearly a Cynipini-focused analysis will be needed to work out generic boundaries within the tribe.

Two monotypic cynipoid taxa were not available for this study, Austrocynipidae and Qwaqwaiini, due to their rarity. Perhaps the more critical of the two are Austrocynipidae, which have also been argued to be the cynipoid with the most plesiomorphic morphological features, including a true pterostigma [15]. Including this taxon could prove important for truly understanding cynipoid evolution—however, based on our results it is likely it may just nest near *Ibalia* and the liopterids along with the other entomophagous cynipoids. Qwaqwaiiini, the other unavailable taxon has been included in a previous phylogenetic analysis [20], where it was indecisively recovered among the ‘basal’ cynipids. Given these previous results, they could either fall into an early branching grade of gall wasps, or be included within the core cynipids, among the herb gallers and woody rosid gallers.

### Instability of Eschatocerini and long branches

One of the more enigmatic cynipoids is *Eschatocerus niger,* the sole member of the tribe Eschatocerini. The morphology of these wasps is extremely apomorphic: species lack mandibles; their cuticle is very thin and pale yellow; the wings have a mere suggestion of the marginal cell; and lastly, they gall *Acacia* and *Prosopis* (Fabaceae) in semi-arid regions of South America. The first inclusion of this taxon in a phylogenetic framework was by Ronquist et al. [20], who did not recover Eschatocerini in a stable location. The same issue was encountered here with UCE data. We recovered the taxon on an extremely long branch, but could not detect any problems with the sequence data. We conclude that the taxon posits an extreme case of autapomorphic evolution, both in terms of morphology and molecular data, resulting in challenges for our current models for phylogenetic reconstruction.

The figure included in Additional file 12 summarizes the unstable nature of Eschatocerini in this dataset. Three positions were routinely recovered based on our analyses: Eschatocerini as sister-group to Figitidae; Eschatocerini as sister-group to Cynipidae s.s.; Eschatocerini nested near Paraulacini and Diplolepidini. Given the apomorphic morphology of Eschatocerini, any of these positions can be defended. None of our results place Eschatocerini nested deeper inside of another clade. In a larger Hymenoptera UCE-based analysis (Blaimer et al. in preparation), Eschatocerini is only recovered among early-branching lineages, as sister-group to Cynipoidea or Cynipidae + Figitidae, suggesting its position is sensitive to the inclusion/exclusion of data and taxa.

Ronquist et al. [20] consistently recovered Eschatocerini as sister-group to Diplolepidini and Pediaspidini; this group, in turn, was sister-group to the remaining Cynipidae. Considering a Southern Hemisphere origin of cynipid lineages postulated by Ronquist et al. [20] and also supported by our data, either the basal position of Eschatocerini as sister-group to all cynipoids, or Eschatocerini being sister-group to figitids are plausible. In either of these two scenarios, the early radiation of cynipoids in the Southern Hemisphere scenario yielded the basis of two major clades: the phytophagous cynipids, and the entomophagous lineages including figitids, ibaliids, and others. If Eschatocerini were present around this split, then we may not have the data available to assign this taxon to either side of this cladogenic event.

### Evolution of cynipoid entomophagy

Parnipinae was found in all analyses to be the earliest branching of a monophyletic entomophagous cynipoid clade (((Parnipinae((Ibaliidae + Liopteridae)(Figitidae))). The subfamily has been regarded as a morphological, and perhaps biological link between cynipids and figitids since its description [51], and our data now brings long-awaited evidence suggesting that not only is Parnipinae a member of the entomophagous cynipoids, but indeed, a key taxon in linking this radiation with the origin of the superfamily itself. The various small subfamilies that are branching early within the figitid clade are all associated with galls in some fashion (Mikeiinae, Plectocynipinae and Thrasorinae, unknown for Pycnostigminae), suggesting a possible retention of this biology not only from the recent ancestor with Parnipinae but also perhaps from a much earlier ancestor with Paraulacini (see discussion above). Relationships between these early figitid lineages were not well-resolved in previous studies [18]. Moreover, our current results are more logical from an evolutionary standpoint than previous phylogenies assuming ibaliids and liopterids as earliest branching lineages [14, 17, 20, 52]. This scenario was challenging to explain due to its implications of early cynipoids being parasitoids of wood boring insect larvae, then diverging to specialized phytophagy (cynipids) in one and entomophagy in another clade (figitids).

Our estimated age of the clade Ibaliidae + Liopteridae with ca. 125 Ma is considerably younger than the estimated 145 Ma age of the same node from Ronquist [15]. We also find here a much younger age for the split of *Paramblynotus* (Oriental) and *Oberthuerella* + *Pseudibalia* (ca. 75 Ma), and between *Pseudibalia* (Neotropical) and *Oberthuerella* (Afrotropical) (ca. 60 Ma), as opposed to Ronquist’s [15] estimate of > 100 Ma. Peters et al. [43] estimated stem-group siricoids, the presumed hosts of proto-ibaliids, to be Triassic in age, suggesting our estimates here are perhaps too young and influenced by restricted taxon sampling.

Our data suggest the subfamilies of Figitidae were established around 125 Ma. As these internal branches are relatively short (Fig. 1b), presumably these lineages diverged quite rapidly. While the relationships among the ‘core’ figitid subfamilies, as recovered here, are remarkably similar to previous results [17, 18], albeit with overall improved support, the early relationships within the figitid clade are quite novel, perhaps as a consequence of the “correct” placement of ibaliids and liopterids. For example, we consistently found Euceroptrinae as sister-group to Liopteridae, but never Euceroptrinae grouping with the other gall-associated figitids [as in 18]. This grouping has never been suggested before, as far as we know, and morphologically, the two groups possess no obvious synapomorphies.

The relationship of Charipinae to core Figitidae has been supported by all previous analyses since Buffington et al. [17], except Ronquist et al. [20] who found charipines and eucoilines sister-group to each other. Charipines are hyperparasitoids (i.e., parasitoids of other parasitoids) of braconids (Ichneumonoidea) and aphelinids (Chalcidoidea) via their aphid hosts, and their placement outside the usually Diptera-associated core-Figitidae has logical appeal considering the evolution of host use. Our data continue to support a perspective previously discussed by Ronquist [15] and Buffington et al. [18] that a critical bridge between the earlier branching gall-associated figitids and the more derived figitids is a period associated with the aphid community.

The last set of relationships we focus on within Figitidae is the (Figitinae, Aspicerinae) + (Emargininae, Eucoilinae) clade, containing the most diverse figitid lineages (Aspicerinae, Figitinae and Eucoilinae) which are all parasitoids of cyclorrhaphan Diptera (flies with a puparium), in cases where host use is known. Aspicerines and figitines have historically been recovered as sister-groups to each other [17, 18, 20] as in our results; the same is true for a sister relationship of Emarginae and Eucoilinae [17, 18]. Within Eucoilinae, the arrangement of tribes presented here reflects the topology in Buffington et al. [17], but not that of Buffington et al. [18]. A more in-depth analysis of Eucoilinae needs to focus on better understanding tribal level relationships within this subfamily. Wiegmann et al. [53] estimated the speciose lineage of cyclorrhaphan flies to be between 145–150 Ma old, much older than the oldest fossil for the group. However, Wiegmann et al.’s [53] estimate aligns with our estimate for figitine and eucoiline early evolution, starting around 125 Ma (Fig. 2), which is considerably older than previous estimates [18, 54; Eucoilinae ca. 80 Ma and Figitinae 100 Ma]. A scenario of host flies emerging and diversifying around 145 Ma, followed by colonization by Figitinae and Eucoilinae, and with subsequent speciation in each parasitoid lineage, appears most likely based on our new estimates. Within Eucoilinae, tribal-level diversification is estimated here at 80 Ma, slightly older than the estimated age of Schizophora [40–60 Ma, 53], the dipteran lineage that the vast majority of Eucoilinae parasitize. This suggests either that early eucoiline wasps must have parasitized proto-schizophoran Diptera, or alternatively, that the origin of Schizophora is older than currently estimated.

### Cynipoid evolution—experimentation followed by specialization

The topology and short internal branch lengths recovered in our analyses suggest a very rapid ‘experimental’ phase of diversification among both entomophagous cynipoids as well as the phytophagous gall inducers. This initial phase was followed by the adaptation and specialization on particular host lineages; however, in figitids, the main catalysts for (co-) diversification appear to be cyclorrhaphan Diptera, and in cynipids, Fagaceae, and in particular the oaks (*Quercus*). Ongoing research on the molecular components of host immune suppression, as well as gall induction, may explain how these two apparently divergent biological strategies can be reconciled in the same phylogeny.

Godfray [10] and Quicke [11] both suggested the molecular mechanisms for gall induction are likely present in the parasitoid genome, co-opted from components used in mitigating a host immune response. Cambier et al. [55] explored the gall induction scenario using transcriptomics, concluding that there are a large number of unique novel genes expressed by cynipid gall wasps but no other Hymenoptera. However, there is a core set of proteins found in the venom glands of both entomophagous and phytophagous cynipoids [Table 4 in 55]. In parasitoid cynipoids, these proteins (and their relatives) are used for host immune suppression [56, 57] and antimicrobial protection [58]; in galling cynipoids, these proteins are presumably used to protect the early instar larva, as well as initiating the breakdown of cell walls in the early stages of gall induction [55, 59]. Gobbo et al. [60] provide the most advanced framework for comparative genomics on this question, and a natural progression of this research would be to include many more gall-inducing and non-gall-inducing Cynipoidea as well as platygastroids.

Considering these shared molecular characteristics in light of our phylogeny and ancestral reconstructions (Figs. 1 and 3), we can postulate the following scenario leading to cynipoid diversity, in particular in Cynipini and Eucoilinae: (1) a common ancestor of Platygastroidea + Cynipoidea possessed the molecular tools that could be used for host immune suppression and potentially also gall induction; (2) a subset of taxa modified this ancestral toolkit to a strategy combining aspects of parasitoidism and galling behavior: inquilinism; (3) gall inducers evolved additional unique hormones for gall growth/host plant manipulation, while parasitoids further diversified their toolkit and host repertoire; (4) this molecular fine-tuning necessitated for both gall induction and host larva manipulation potentially led to high species diversity in Cynipini on oaks, and in Eucoilinae on Diptera.

Cynipoids are not alone in having both gall-inducing and parasitoid members: the Chalcidoidea is another such group exhibiting both strategies. LaSalle [61] first suggested that galling in Chalcidoidea has arisen independently many more times than in any other hymenopteran group (perhaps, even across all insects). Heraty et al. [7] and Peters et al. [62] later provided the phylogenetic evidence for the simultaneous independent evolution of derived galling behavior in Chalcidoidea that was lacking in LaSalle [61]. While there is some evidence that galling chalcidoids evolved from phytophagous or seed-feeding ancestors, the vast majority are hypothesized to have evolved from parasitoids of galling insects [61, 63, 64]. Hence, the patterns that explain the evolution of phytophagous and entomophagous cynipoids and chalcidoids may also explain similar life history patterns in other arthropods.

## Conclusions

Using comprehensive ingroup and outgroup sampling, our study has clarified certain hypotheses in cynipoid evolution, as outlined above. First, we were able to firmly place the wood-boring parasitoid family Ibaliidae, previously considered an early-branching cynipoid, as a close relative to Figitidae in a larger Figitidae s.l. clade. By contrast, as earliest diverging lineages in our analyses emerged the cynipid tribes Paraulacini, a lineage of inquilines or parasitoids in galls on *Nothofagus,* and Diplolepidini, a group of gall-making wasps on Rosaceae. As a consequence, the family Cynipidae is rendered paraphyletic and shown to not have a reciprocally monophyletic sister-group relationship with Figitidae as previously assumed. Lastly, we found support for the agastoparasitism hypothesis [13], with the inquilinous genera *Synophromorph*a and *Periclistus* being close relatives of their host genus *Diastrophus.* Our ancestral state reconstructions have favored either a parasitoid-first scenario, or an inquiline-first scenario for Cynipoidea, depending on whether a simple or more complex state model is used, and whether Paraulacini are considered inquilines or parasitoids. In any case, our analyses indicate that the gall-making behavior is derived and evolved multiple times independently in Cynipoidea. Overall, it appears that the diversity of species and life history strategies we see across Cynipoidea today is both the result of radical innovation, such as switching from inquilinism to parasitoidism and gall-making (or vice versa), and of successful specialization to new hosts, be they plants or other insects. By reconstructing a robust phylogeny and highlighting patterns of life histories, our study has established the framework for further physiological and comparative genomic work between gall-making, inquiline and parasitoid lineages in this fascinating system, and will have many implications for the evolution of parasitism and other life histories in Hymenoptera.

## Methods

### Taxon sampling

We selected 119 taxa across all major lineages with the superfamily Cynipoidea. Specimens included in this global study were collected in accordance with local regulations and necessary permits. All specimens were pulled from the accessioned collections at the United States National Museum of Natural History (USNM), Smithsonian Institution, and represent all cynipoid lineages save for the very rare Austrocynipidae and Qwaqwaiini (Cynipidae). Individuals were chosen from series of at least eight individuals from the same collecting event; one specimen was used for destructive DNA extraction (below) while the other specimens were kept as a voucher series. Specimens collected within the past twenty years were chosen first, but older specimens (oldest dating to 1935) were also included when younger specimens were not available. Additional file 1 contains voucher information for all taxa and further specimen data. We also included seven taxa from other Hymenoptera lineages with varying degree of relatedness to cynipoid wasps: four platygastroid taxa (*Sparasion cullaris*, *Nixonia watshami*, *Trissolcus* sp. and *Platygaster* sp.), one species of Eulophidae (*Leptocybe invasa*), one species of Diapriidae (*Propsilomma columbianum*) and one braconid species (*Callihormius bifasciatus*). UCE sequences for two taxa from a previously published Hymenoptera data set [65] were further included in our analyses.

### UCE data collection

We extracted genomic DNA destructively or non-destructively (specimen retained after extraction) from whole specimens using the DNeasy Blood and Tissue Kit (Qiagen, Valencia, CA, USA). We quantified genomic DNA for each sample using a Qubit fluorometer (High sensitivity kit, Life Technologies, Inc., Carlsbad, CA). Between < 5 ng and 567 ng DNA was sheared for 0–60 secs (amp = 25, pulse = 10) to a target size of approximately 500–600 bp by sonication (Q800, Qsonica Inc., Newtown, CT) and used as the input for a modified genomic DNA library preparation protocol (Kapa Hyper Prep Library Kit, Kapa Biosystems, Wilmington, MA) that incorporated “with-bead” cleanup steps [66] and a generic SPRI substitute [67]. We used TruSeq-style adapters during adapter ligation [68]. Libraries had post-PCR concentrations from 0.1–31.6 ng/µL. We combined groups of eight to ten libraries at equimolar ratios and enriched each pool using a set of custom-designed probes (MYcroarray, Inc., now ArborBiosciences, Ann Arbor, MI) targeting 2590 UCE loci in Hymenoptera [32] and now sold as predesigned panel “myBaits UCE Hymenoptera 2.5Kv2P”. We followed target enrichment procedures for the MYcroarray MYBaits kit V2 [69], except we used a 0.2X concentration of the standard MYBaits concentration and added 0.7 µL of 500 µM custom blocking oligos designed against our custom sequence tags. We ran the hybridization reaction for 24 h at 65 °C, subsequently bound all pools to streptavidin beads (Dynabeads MyOne Streptavidin T1; Life Technologies, Inc., Carlsbad, CA) and washed bound libraries according to a standard target capture protocol [69].

We used the with-bead approach for PCR recovery of enriched libraries as described in Faircloth et al. [70], and performed qPCR library quantification and combined enriched pools at equimolar concentrations into final pools (including 96–100 individual samples) based on the estimated size-adjusted concentrations. Final pools were analyzed on a TapeStation (Agilent Technologies, Santa Clara, CA) and size-selected for 250–600 bp with a BluePippin (SageScience, Beverly, MA). All of the UCE laboratory work was conducted in and with support of the Laboratories of Analytical Biology (L.A.B.) facilities of the National Museum of Natural History, Smithsonian Institution, in Washington, DC. The pooled libraries were sequenced using several lanes of 125-bp paired-end sequencing on an Illumina HiSeq 2500 instrument at the University of Utah’s Huntsman Cancer Institute. Library concentration and sequencing statistics are summarized in Additional file 2.

### Processing and alignment of UCE data

Data processing relied on scripts within the PHYLUCE package v1.5 (Faircloth 2016). Demultiplexed FASTQ data was trimmed for adapter contamination and low-quality bases using Illumiprocessor [71], based on the package Trimmomatic [72]. We assembled the cleaned reads using the phyluce_assembly_assemblo_trinity.py wrapper around the program Trinity (version trinityrnaseq_r20140717) [73]. Species-specific contig assemblies were aligned to a FASTA file of all enrichment baits (script *phyluce_assembly_match_contigs_to_probes*, with settings min_coverage = 50, min_identity = 80), creating a relational database containing the matched probes. We used *phyluce_assembly_get_match_counts.py* to generate a list of UCE loci shared across all taxa, which was then used to create separate FASTA files for each UCE locus containing sequence data for taxa present at that particular locus, using *phyluce_assembly_get_fastas_from_match_counts.py*. We aligned sequence data for each locus using MAFFT [74] and trimmed our alignment using Gblocks [75; with the following relaxed settings: b1 = 0.5, b2 = 0.5, b3 = 12, b4 = 7], using the relevant PHYLUCE scripts. Sequence quality statistics were calculated for adapter-trimmed reads, Trinity contigs and UCE contigs using *phyluce_assembly_get_fastq_lengths*. We selected a 50%, 60% and 70% taxon complete set of loci (containing loci with alignment data from at least 63, 75 and 88 of 126 taxa, and retaining 1147, 918 and 626 UCE loci for analysis, respectively) for further analyses using the script *phyluce_align_get_only_loci_with_min_taxa*. For concatenated phylogenetic analyses, we combined individual alignments of UCE loci into one nexus alignment file with *phyluce_align_format_nexus_files_for_raxml.py* for subsequent phylogenetic analyses.

### Phylogenetic inference

*Analyses based on concatenated data sets.* We used the program AMAS v1.0 [76] to calculate several alignment statistics, e.g., alignment length, amount of missing data, number of parsimony-informative sites (PIC), and base composition for the 50%, 60% and 70% matrices (Additional file 3). We partitioned the data matrices using the Sliding-Window Site Characteristics (SWSC-EN) algorithm described in Tagliacollo and Lanfear [77]. This algorithm has been designed specifically to model patterns of rate variation within and among UCE loci by dividing loci into core and flanking regions. We subsequently used PartitionFinder2 [78] and the *r cluster* algorithm [79] to combine subsets with similar properties and select the best-fitting model of evolution (selection limited to GTR, GTR + G, or GTR + G + I). We analyzed the resulting concatenated data matrices with 2793 (50%), 743 (60%) and 571 (70%) partitions, as well as unpartitioned versions with Maximum Likelihood (ML) best-tree and bootstrap searches (N = 1000) in IQ-TREE v1.6 [80]. For unpartitioned data sets, we let ModelFinder in IQ-TREE select the best-fitting model of evolution preceding analyses. Analyses were rooted using the outgroup method and our outer outgroup taxon *Callihormius bifasciatus* (Braconidae). We further estimated gene trees for individual UCE loci using IQ-TREE under partitioning schemes estimated with the SWSC-EN algorithm. We reconstructed gene trees only from 1143 UCE loci present in the 50% taxon completeness set that had an alignment length ≥ 200 nucleotides. The resulting ML best trees with branch lengths were then used to perform coalescent species-tree analysis in ASTRAL-III v5.6.3 [81]. We chose not to perform statistical binning [82] prior to gene tree estimation due to concerns about this procedure [83].

### Sensitivity analyses

Due to major differences in higher-level relationships within Cynipoidea recovered in our analyses compared to a previous analysis [20], we tested the influence of outgroup rooting by removing all non-cynipoid taxa from the 50%, 60% and 70% data set and repeating ML analyses while rooting on the ibaliid branch with *Ibalia anceps* [as in 20]. We also performed analyses on this data set excluding the non-cynipoid outgroups and rooting with *Paraulax queulensis*. We then tested whether the likelihoods of the two pairs of trees differed significantly from each other by performing a Shimodaira-Hasegawa (SH) test in IQ-TREE using 1000 RELL replicates.

Given the relatively long branch length and isolated position of *Eschatocerus niger* (Eschatocerini) in the above analyses, we investigated the possibility of interference of this taxon with the placement of other taxa due to long-branch attraction. We therefore repeated ML analyses while excluding this taxon (unpartitioned 50% and 70% matrices only). We investigated GC content and GC variance as another potential factor leading to uncertainty in reconstructing the phylogenetic position of *E. niger*, since GC content and GC variance across UCE loci and taxa has been found to bias phylogenetic inference [84]. We used AMAS [76] to calculate GC content per taxon across the 50% completeness matrix, and also calculated GC content for each UCE locus. After initial exploration showed that GC content did not vary much across taxa, we focused our efforts specifically on analyzing the impact of varying GC content across loci on phylogenetic inference and the position of *E. niger*. We sorted the 1147 UCE loci into 10 bins of each 114–115 loci based on their GC content (e.g., bin 1 contained the 114 loci with lowest GC content, bin 10 the 114 loci with highest GC content), concatenated loci in each bin and performed ML analyses. These bins and their GC content are defined in Additional file 4. We performed ML analysis on each of these ten data sets, and scored the support and position of *E. niger* in the resulting trees.

### Divergence time estimation

We estimated time-calibrated phylogenies using information from five fossils within the Figitidae and Cynipidae (Additional file 5): *Protimaspis costalis* (assigned to stem-group Cynipini) [85], *Diplolepis vetus* (assigned to stem-group *Diplolepis*) [85], *Palaeoaspicera orientalia* (assigned to stem-group Aspicerinae) [18], *Syneucoila magnifica* (assigned to stem-group Eucoilinae) [54] and *Rovnoeucoila tympanomorpha* (assigned to stem-group Ganaspini) [54]. We further obtained three different age ranges (as minimum and maximum ranges) for the root of the phylogeny by summarizing over divergence ages estimated in Peters et al. [43]. We used the 95% maximum age range (196–273 Ma), the 95% minimum age range (181–246 Ma), and the median age range (211–236 Ma) across four estimates from that study [43] for the split of Cynipoidea with Platygastroidea, and implemented these as minimum and maximum bounds on the root node. We pruned all but two closest outgroups (*Platygaster sp*. and *Sparasion cullaris*), as well as eight taxa with very little sequence divergence to their closest relative from the tree and alignment prior to divergence time estimation.

We employed approximate likelihood to estimate divergence times in mcmctree and codeml as included in PAMLv4.9 [86], using the 50% completeness matrix and the best maximum likelihood tree resulting from SWSC-EN partitioning. Approximate likelihood calculation is a two-step process consisting first of branch length estimation by maximum likelihood, together with the gradient and Hessian of the likelihood function at the maximum likelihood estimates. Divergence times are then estimated in a second step using MCMC and the gradient and Hessian to construct an approximation to the likelihood function. For each of the three root calibration ranges (employed as soft minimum and soft maximum age), we set up four independent runs using the independent-rates models and standard parameters. The five fossil calibrations were implemented as soft minima using default settings (truncated Cauchy distributions with an offset of 0.1, a scale parameter of 1 and a left tail probability of 0.025). After several trials with different values for burnin, sample frequency and number of samples, we achieved convergence implementing *burnin* = 500,000, *samplefreq* = 10, and *nsamples* = 1,000,000. We visualized mcmc convergence and effective sample sizes using Tracer v1.7.1 [87] and summarized across all results. To test the impact of our calibrations we also performed analyses without sequence data using only the prior, and we investigated the influence of each individual calibration on posterior estimates by performing analyses that sequentially excluded each of the five calibrations (performed using the median root range only).

### Evolution of cynipoid life histories

Since we were interested in the evolutionary trajectory of cynipoid life histories, we inferred ancestral states using our dated phylogeny. We performed two different sets of reconstructions, varying the level of detail as to how we subdivided cynipoid life history states, with states reflecting those used in Ronquist et al. [20]. First, we scored all ingroup taxa (N = 111) and the two outgroup taxa represented in the dated phylogeny according to the three characters states *parasitoid*, *inquiline*, and *galler* (= three-state model). In a second, separate set of analyses (= seven-state model), we further subdivided the gallers according to their host plant to reconstruct the ancestral history of galling, thus analyzing the following seven states: *parasitoid*, *inquiline*, *galler on Fagaceae*, *galler on herbs*, *galler on Acer*, *galler on Rosaceae*, and *galler on Acacia*.

Since a main point of interest was to reconstruct the life history of the “ancestral cynipoid”, we performed all analyses while both including and excluding the two non-cynipoid outgroup taxa. Biological evidence on the life history strategy of ten taxa was only anecdotal, although these are all considered parasitoids by experts. To ensure that coding these taxa as parasitoids would not have a misleading effect on our reconstructions overall, we performed two different variant analyses in which we either coded these taxa as parasitoids or as “unknown” (i.e. as “?”). Moreover, we coded the three taxa belonging to the tribe Paraulacini selectively either as parasitoids or inquilines, since their life history remains unclear and they may exhibit an intermediate strategy by not only usurping the host gall, but also killing the host in the process (i.e. are “lethal inquilines” sensu Nieves-Aldrey [40]). Altogether, we thus performed eight reconstructions with slightly varying trait compositions within each of the two main sets. The two main character matrices are included in Additional file 1. For ancestral state reconstruction (ACR), we used the rayDISC function in the package corHMM in R v4.0.0 (https://www.R-project.org/) which can analyze multivariate traits, and our main time-calibrated phylogeny estimated while employing the median range calibration on the root node. We performed ACR using all three models available in corHMM, ‘equal rates’ (ER, one transition rate), ‘symmetric’ (SYM, forward and reverse transition have the same rate) and ‘all rates different’ (ARD), and compared the fit of these models using a likelihood ratio test (LHT) on the resulting –lnL scores. We performed all analyses twice with the same settings to ascertain robustness of reconstructions.

## Supplementary information

**Additional file 1.** Information on specimen vouchers and collection data, as well as traits. Table listing USNMENT voucher numbers for newly sequenced taxa, collection data as available, and character states for the life history analyses. Character states are: 3-state model: 0 = parasitoids, 1 = inquilines, 2 = gallers; 7-state model: 0 = parasitoids, 1 = inquilines, 2 = gallers-Fagaceae, 3 = gallers-herbs, 4 = gallers-Acer, 5 = gallers-Rosaceae, 6 = gallers-Acacia; taxa with unspecified states were not included in dating and ancestral reconstructions; taxa with two states indicated were coded both ways in two separate analyses. n/a = not applicable, n.a. = not available; Biogeographic regions are: AFR = Afrotropical, AUS = Australasian, NEA = Nearctic, NEO = Neotropical, ORI = Oriental/Indomalayan, PAL = Palearctic.

**Additional file 2.** Library preparation and UCE sequence capture statistics. Table listing for each taxon included in the study the pre-library preparation DNA concentration, the total DNA input, the post-library preparation DNA concentration, the total raw read count, the total number of assembled contigs and mean length, and the number of assembled UCE contigs and their mean length, the number of UCE loci, and average GC content.

**Additional file 3.** Alignment statistics. Various alignment statistics calculated with AMAS (Borowiec 2016).

**Additional file 4.** BINs defined for GC content experiments. Table listing average, minimum and maximum GC content across loci, topology estimated using unpartitioned ML analysis (for a sketch of topology A,B,C refer to Additional File 12), and support for the position of *E. niger*.

**Additional file 5.** Calibration points used in this study and their justification. Table outlining details for each fossil calibration used in our study.

**Additional file 6.** Additional trees estimated from partitioned concatenated analyses. All trees are presented as cladograms for clarity of relationships, and are based on a combined ML search for the best tree and 1000 bootstrap replicates. Bootstrap support values are displayed next to respective nodes. Analyses were rooted using the outer outgroup *Callihormius bifasciatus*. **A)** 60% completeness matrix using SWSC-EN partitioning scheme, **B)** 70% completeness matrix.

**Additional file 7.** Additional trees estimated from unpartitioned concatenated analyses. All trees are presented as cladograms for clarity of relationships, and are based on a combined ML search for the best tree and 1000 bootstrap replicates. Bootstrap support values are displayed next to respective nodes. Analyses were rooted using the outer outgroup *Callihormius bifasciatus*. **A)** 50% completeness matrix, **B)** 60% completeness matrix, **C)** 70% completeness matrix.

**Additional file 8.** Species Tree estimated with ASTRAL-III. Cladogram estimated using ASTRAL-III coalescent analysis from 1143 UCE gene trees reconstructed with IQTREE. Support values are local posterior probabilities, which are branch support values that measure the support for a quadripartition, not a bipartition.

**Additional file 9.** Summary of topology for ingroup-only analyses and previous hypotheses. **A**. Sketch of the topology recovered when excluding the seven non-cynipoid outgroup taxa from the analyses and rooting with *Ibalia anceps*. Summarized here are identical topologies resulting from unpartitioned Maximum Likelihood analyses with IQ-TREE (combined ML search for best tree and 1000 bootstraps) of 50%, 60% and 70% completeness matrices including 119 ingroup taxa. Most nodes have bootstrap support = 100; ranges represent variation in support across analyses. **B**: Sketch of the main topology recovered by Ronquist et al. (2015). **C**: Sketch of main topology recovered by Buffington et al. (2012).

**Additional file 10.** Additional trees estimated from ingroup-only analyses. All trees are presented as cladograms for clarity of relationships, and are based on a combined ML search for the best tree and 1000 bootstrap replicates using unpartitioned data matrices**.** Bootstrap support values are displayed next to respective nodes. Analyses were rooted with *Ibalia anceps* and excluded non-cynipoid outgroups. **A)** 50% completeness matrix, **B)** 60% completeness matrix, **C)** 70% completeness matrix.

**Additional file 11.** Additional trees estimated while excluding *Eschatocerus niger*. All trees are presented as cladograms for clarity of relationships, and are based on a combined ML search for the best tree and 1000 bootstrap replicates using unpartitioned data matrices**.** Bootstrap support values are displayed next to respective nodes. Analyses were rooted using the outer outgroup *Callihormius bifasciatus*. **A)** 50% completeness matrix, **B)** 70% completeness matrix.

**Additional file 12.** Summary of phylogenetic positions for *Eschatocerus niger*. **A**) *Eschatocerus niger* grouping as sister to Figitidae, **B**) *Eschatocerus niger* grouping as sister to Cynipidae *s.s*., **C**) *Eschatocerus niger* grouping as sister to Figitidae + (Cynipidae *s.s* + (Diplolepidini + Pediaspidini)). Topology A is supported by all analyses on the full data set (all concatenated ML analyses and ASTRAL-III coalescent analysis) as well as four data subsets binned by GC content. Topology B is supported by four of the GC bins, while Topology C is supported by two of the GC bins. For details refer to Additional file 4.

**Additional file 13.** Results from Divergence dating analyses. Summarized are median ages and 95% HPD intervals for 8 separate sets of MCMCTREE analyses, implementing different root calibrations and calibration exclusions. Node numbers refer to Fig. 2.

**Additional file 14.** Ancestral state reconstructions of cynipoid life histories using a three-state model. Reconstructions were performed under the ARD-model using the rayDISC function in the R-package corHMM. Summarized here are ancestral state probabilities (ranging from 0–1) from 8 variations of analyses on the three-state model, with states 0 = parasitoid, 1 = inquiline and 2 = galler. Variations are a-i = Outgroups included, Paraulacini are coded as parasitoids, uncertain taxa coded as parasitoids; a-ii = Outgroups included, Paraulacini are coded as inquilines, uncertain taxa coded as parasitoids; b-i = Outgroups excluded, Paraulacini are coded as parasitoids, uncertain taxa coded as parasitoids; b-ii = Outgroups excluded, Paraulacini are coded as inquilines, uncertain taxa coded as parasitoids; c-i = Outgroups included, Paraulacini are coded as parasitoids, uncertain taxa coded as unknown; c-ii = Outgroups included, Paraulacini are coded as inquilines, uncertain taxa coded as unknowns; d-i = Outgroups excluded, Paraulacini are coded as parasitoids, uncertain taxa coded as unknowns; d-ii = Outgroups excluded, Paraulacini are coded as inquilines, uncertain taxa coded as unknowns. For each node, the state with the highest likelihood has been bolded.

**Additional file 15.** Ancestral state reconstructions of cynipoid life histories using a seven-state model. Reconstructions were performed under the ER-model using the rayDISC function in the R-package corHMM. Summarized here are ancestral state probabilities (ranging from 0–1) from 8 variations of analyses on the seven-state model, with states 0 = parasitoid, 1 = inquiline and 2 = galler-Fagaceae, 3 = galler-herbs, 4 = galler-Acer, 5 = galler-Rosaceae, 6 = galler-Acacia. Variations are a-i = Outgroups included, Paraulacini are coded as parasitoids, uncertain taxa coded as parasitoids; a-ii = Outgroups included, Paraulacini are coded as inquilines, uncertain taxa coded as parasitoids; b-i = Outgroups excluded, Paraulacini are coded as parasitoids, uncertain taxa coded as parasitoids; b-ii = Outgroups excluded, Paraulacini are coded as inquilines, uncertain taxa coded as parasitoids; c-i = Outgroups included, Paraulacini are coded as parasitoids, uncertain taxa coded as unknown; c-ii = Outgroups included, Paraulacini are coded as inquilines, uncertain taxa coded as unknowns; d-i = Outgroups excluded, Paraulacini are coded as parasitoids, uncertain taxa coded as unknowns; d-ii = Outgroups excluded, Paraulacini are coded as inquilines, uncertain taxa coded as unknowns. For each node, the state with the highest likelihood has been bolded. Grey shaded fields highlight nodes with conflict or variable support between the variations of analyses.

## Data Availability

The datasets supporting the conclusions of this article are available in the NCBI Sequence Read Archive repository under Bioproject accession PRJNA647791 https://www.ncbi.nlm.nih.gov/sra/PRJNA647791, and in the Dryad repository under accession https://doi.org/10.5061/dryad.tx95x69w6 [88].

## Abbreviations

ACR: Ancestral state reconstruction
AHE: Anchored hybrid enrichment
ARD: All rates different
BS: Bootstrap support
bp: Base pairs
ER: Equal rates
HPD: Highest posterior density
LHT: Likelihood ratio test
LPP: Local posterior probability
Ma: Million years ago
MCMC: Markov Chain Monte Carlo
MCC: Maximum clade credibility
ML: Maximum likelihood
MRCA: Most recent common ancestor
MY: Million years
P: Probability
SI/HPC: Smithsonian Institution high performance cluster
SH: Shimodaira-Hasegawa
s.l.: Sensu lato
s.s.: Sensu stricto
SWSC-EN: Sliding-Window Site Characteristics Entropy
SYM: Symmetric
UCEs: Ultraconserved elements
USNM: United States National Museum of Natural History
