# Supplementary material for: Comprehensive phylogenomic analyses re-write the evolution of parasitism within cynipoid wasps
Source: BMC Evol Biol. 2020 Nov 23;20:155. doi: 10.1186/s12862-020-01716-2 (PMC7686688; doi:10.1186/s12862-020-01716-2)
Supplement: Supplementary file 8 — Additional file 8. Species Tree estimated with ASTRAL-III. Cladogram estimated using ASTRAL-III coalescent analysis from 1143 UCE gene trees reconstructed with IQTREE. Support values are local posterior probabilities, which are branch support values that measure the support for a quadripartition, not a bipartition. [file 12862_2020_1716_MOESM8_ESM.pdf]

Additional files *for Blaimer et al: Comprehensive phylogenomic analyses re-write the evolution of parasitism within cynipoid wasps. BMC Evolutionary Biology.*

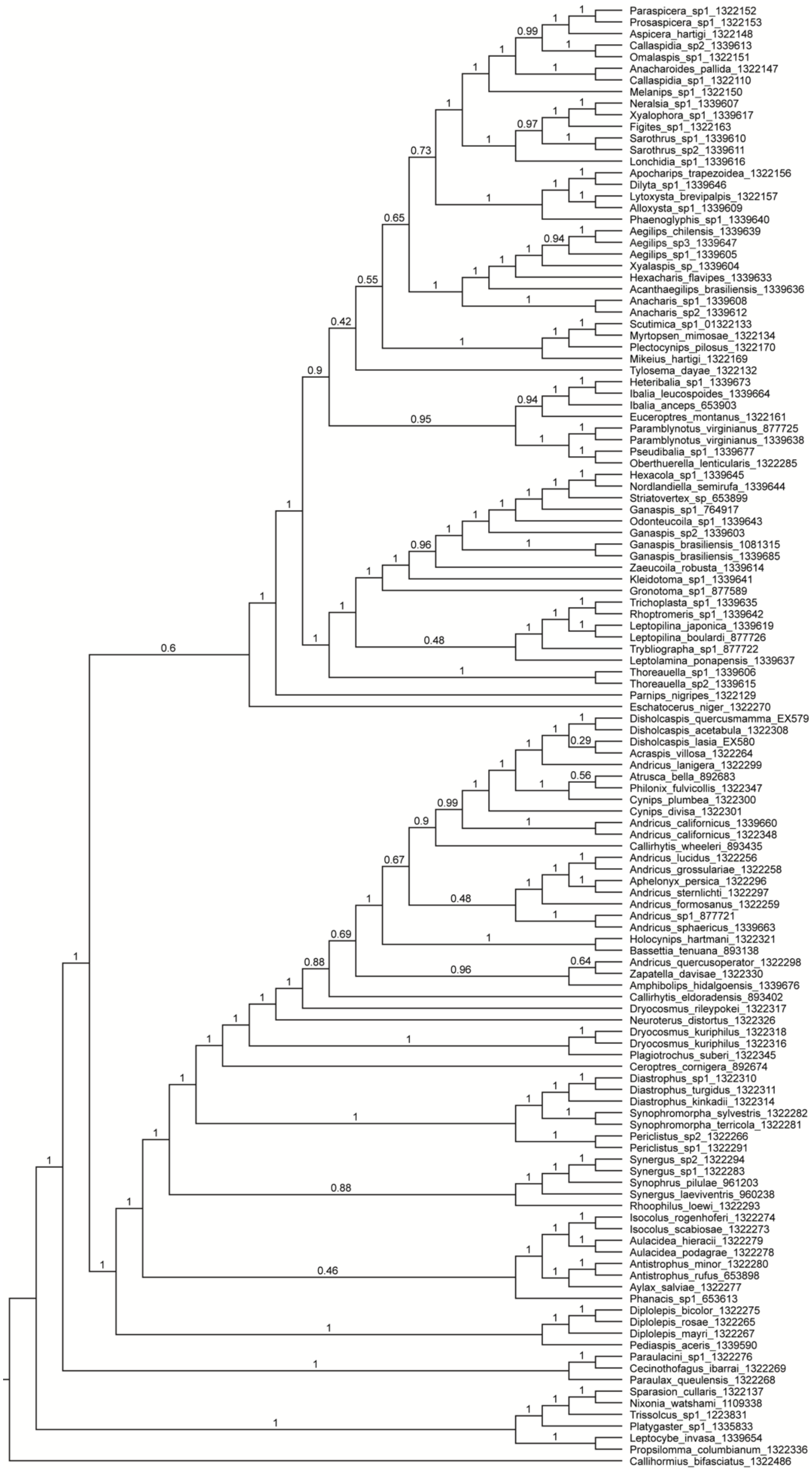

Additional file 8: Species Tree estimated with ASTRAL-III. Cladogram estimated using ASTRAL-III coalescent analysis from 1143 UCE gene trees reconstructed with IQTREE. Support values are local posterior probabilities, which are branch support values that measure the support for a quadripartition, not a bipartition.
