# Supplementary material for: Comprehensive phylogenomic analyses re-write the evolution of parasitism within cynipoid wasps
Source: BMC Evol Biol. 2020 Nov 23;20:155. doi: 10.1186/s12862-020-01716-2 (PMC7686688; doi:10.1186/s12862-020-01716-2)
Supplement: Supplementary file 9 — Additional file 9. Summary of topology for ingroup-only analyses and previous hypotheses. A. Sketch of the topology recovered when excluding the seven non-cynipoid outgroup taxa from the analyses and rooting with Ibalia anceps. Summarized here are identical topologies resulting from unpartitioned Maximum Likelihood analyses with IQ-TREE (combined ML search for best tree and 1000 bootstraps) of 50%, 60% and 70% completeness matrices including 119 ingroup taxa. Most nodes have bootstrap support = 100; ranges represent variation in support across analyses. B: Sketch of the main topology recovered by Ronquist et al. (2015). C: Sketch of main topology recovered by Buffington et al. (2012). [file 12862_2020_1716_MOESM9_ESM.pdf]

Additional files *for Blaimer et al: Comprehensive phylogenomic analyses re-write the evolution of parasitism within cynipoid wasps. BMC Evolutionary Biology.*

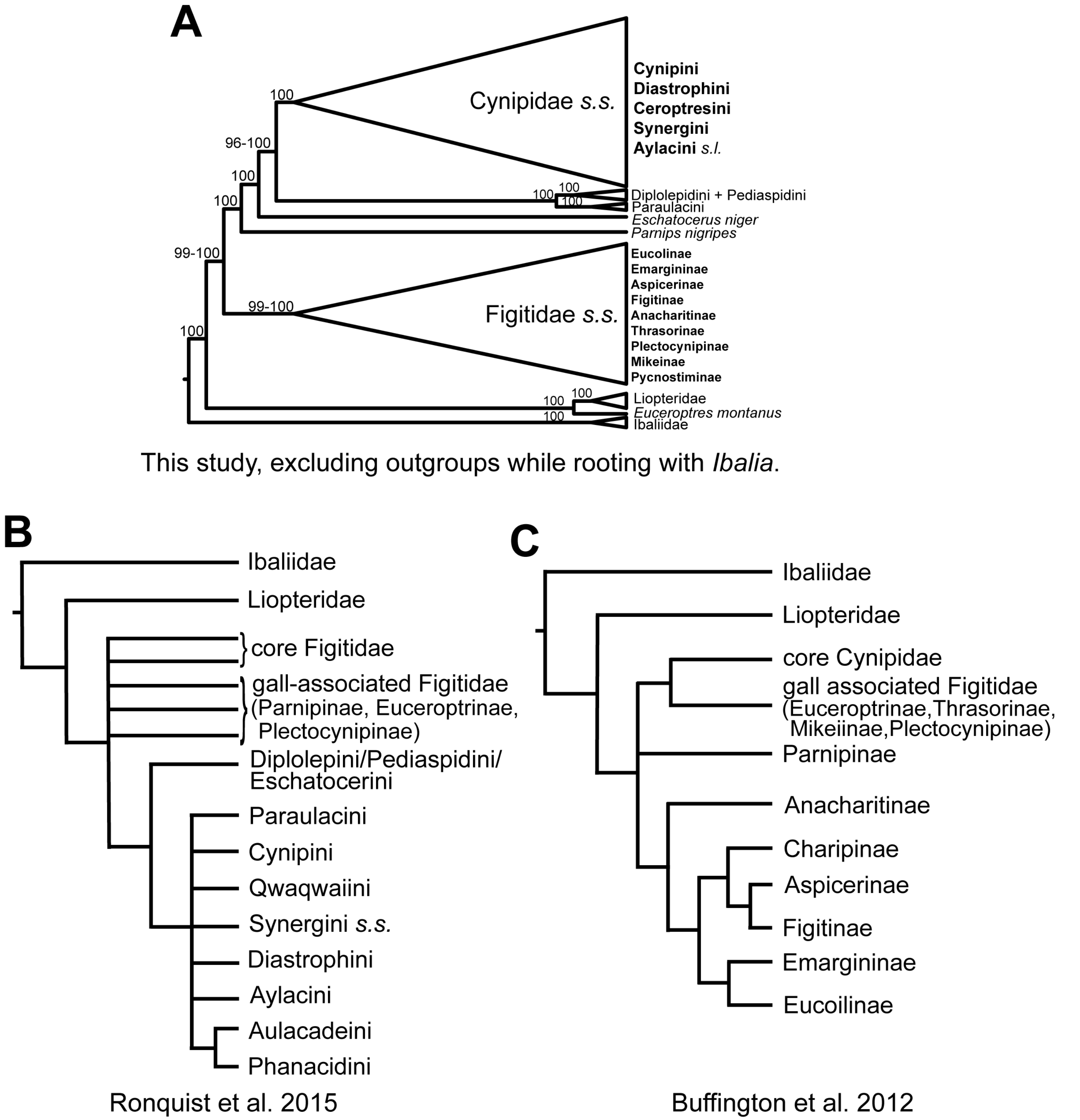

Additional file 9: Summary of topology for ingroup-only analyses and previous hypotheses.
A) Sketch of the topology recovered when excluding the seven non-cynipoid outgroup taxa from the analyses and rooting with *Ibalia anceps*. Summarized here are identical topologies resulting from unpartitioned Maximum Likelihood analyses with IQ-TREE (combined ML search for best tree and 1000 bootstraps) of 50%, 60% and 70% completeness matrices including 119 ingroup taxa. Most nodes have bootstrap support=100; ranges represent variation in support across analyses. **B**) Sketch of the main topology recovered by Ronquist et al. (2015). **C**) Sketch of main topology recovered by Buffington et al. (2012).
