# Supplementary material for: Comprehensive phylogenomic analyses re-write the evolution of parasitism within cynipoid wasps
Source: BMC Evol Biol. 2020 Nov 23;20:155. doi: 10.1186/s12862-020-01716-2 (PMC7686688; doi:10.1186/s12862-020-01716-2)
Supplement: Supplementary file 10 — Additional file 10. Additional trees estimated from ingroup-only analyses. All trees are presented as cladograms for clarity of relationships, and are based on a combined ML search for the best tree and 1000 bootstrap replicates using unpartitioned data matrices. Bootstrap support values are displayed next to respective nodes. Analyses were rooted with Ibalia anceps and excluded non-cynipoid outgroups. A) 50% completeness matrix, B) 60% completeness matrix, C) 70% completeness matrix. [file 12862_2020_1716_MOESM10_ESM.pdf]

***Additional files** for Blaimer et al: Comprehensive phylogenomic analyses re-write the evolution of parasitism within cynipoid wasps. BMC Evolutionary Biology.*

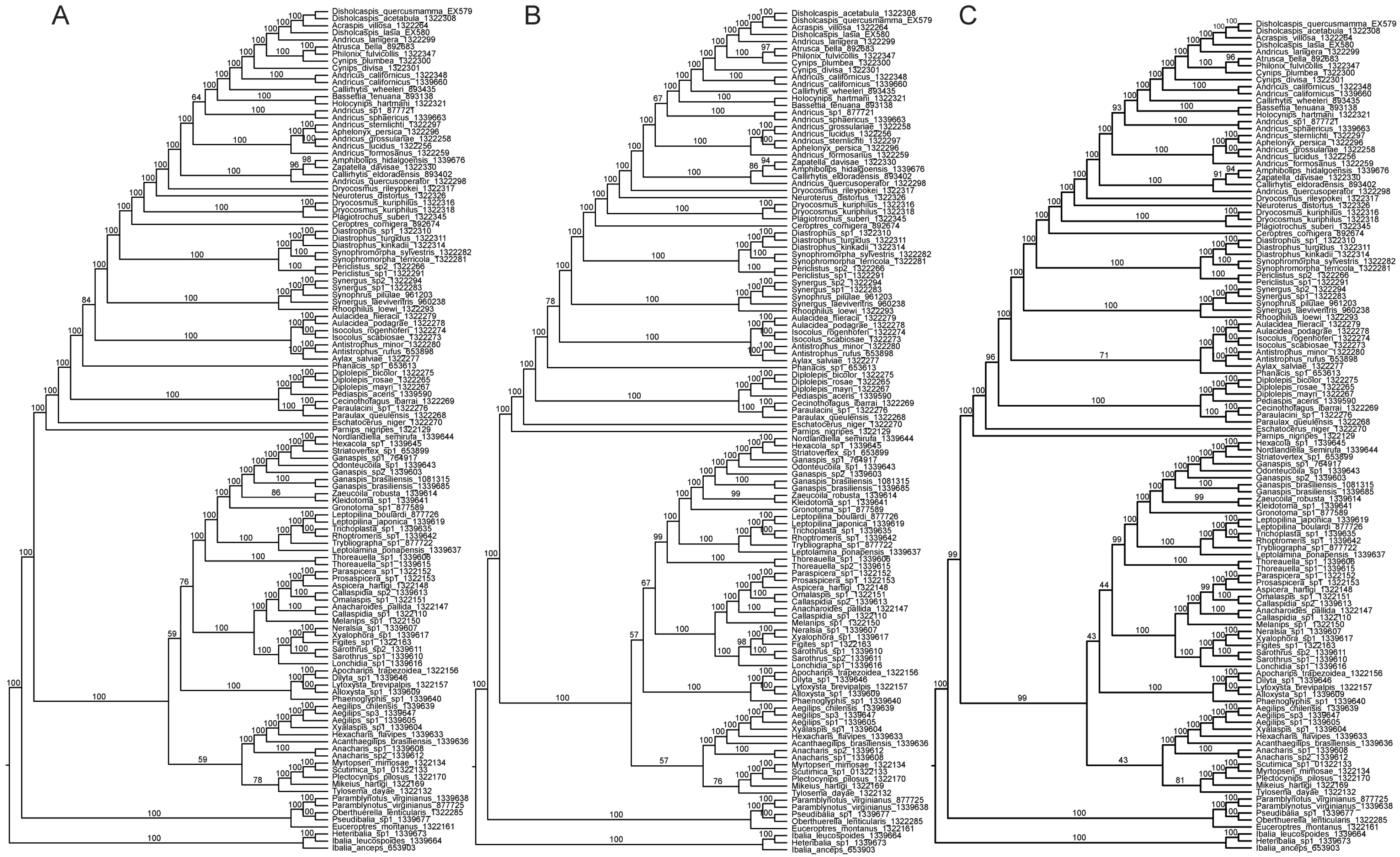

Additional file 10: Additional trees estimated from ingroup-only analyses. All trees are presented as cladograms for clarity of relationships, and are based on a combined ML search for the best tree and 1000 bootstrap replicates using unpartitioned data matrices**.** Bootstrap support values are displayed next to respective nodes. Analyses were rooted with *Ibalia anceps* and excluded non-cynipoid outgroups. **A)** 50% completeness matrix, **B)** 60% completeness matrix, **C)** 70% completeness matrix.
