# Supplementary material for: Comprehensive phylogenomic analyses re-write the evolution of parasitism within cynipoid wasps
Source: BMC Evol Biol. 2020 Nov 23;20:155. doi: 10.1186/s12862-020-01716-2 (PMC7686688; doi:10.1186/s12862-020-01716-2)
Supplement: Supplementary file 11 — Additional file 11. Additional trees estimated while excluding Eschatocerus niger. All trees are presented as cladograms for clarity of relationships, and are based on a combined ML search for the best tree and 1000 bootstrap replicates using unpartitioned data matrices. Bootstrap support values are displayed next to respective nodes. Analyses were rooted using the outer outgroup Callihormius bifasciatus. A) 50% completeness matrix, B) 70% completeness matrix. [file 12862_2020_1716_MOESM11_ESM.pdf]

Additional files *for Blaimer et al: Comprehensive phylogenomic analyses re-write the evolution of parasitism within cynipoid wasps. BMC Evolutionary Biology.*

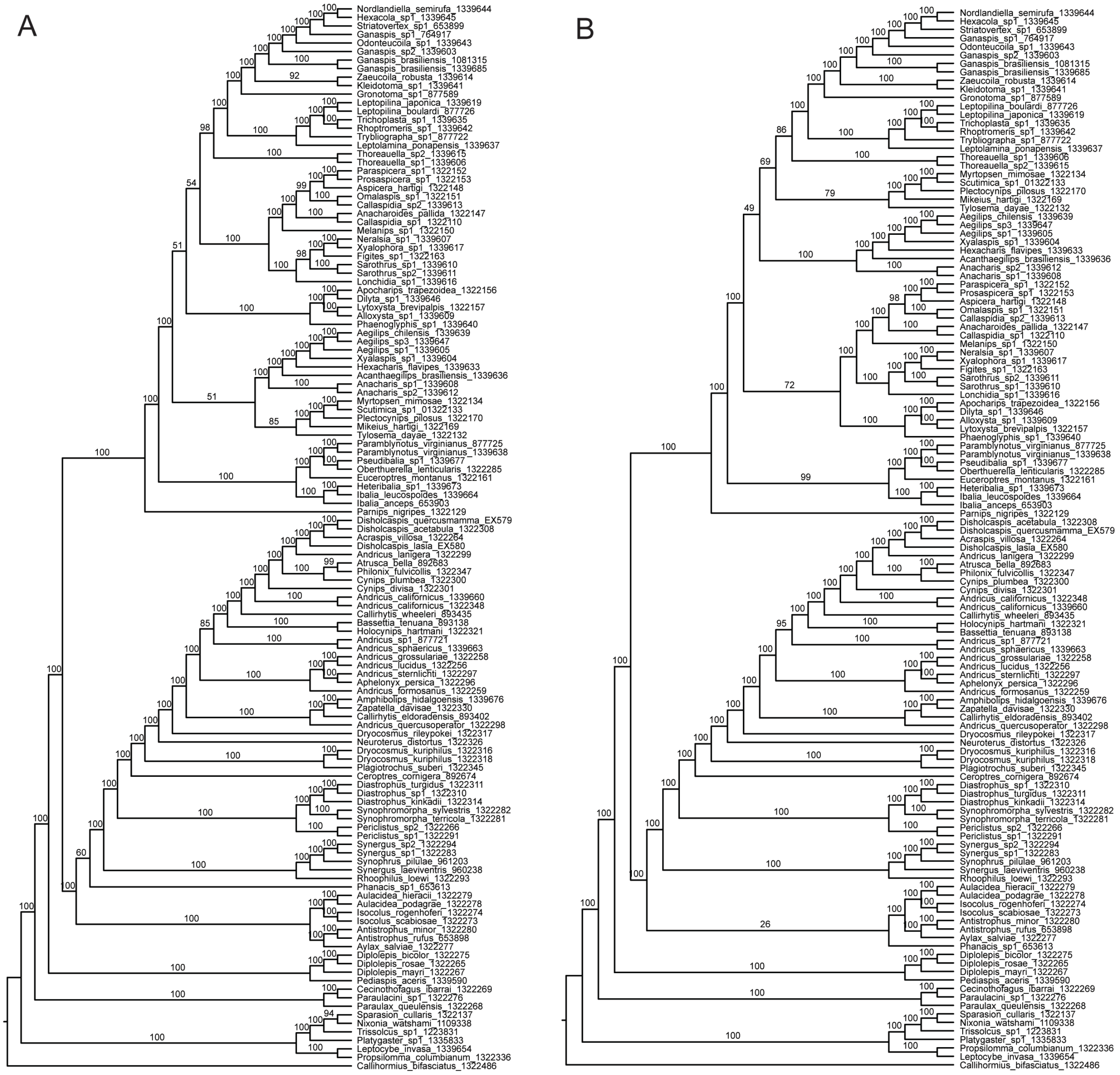

Additional file 11: Additional trees estimated while excluding *Eschatocerus niger*. All trees are presented as cladograms for clarity of relationships, and are based on a combined ML search for the best tree and 1000 bootstrap replicates using unpartitioned data matrices**.** Bootstrap support values are displayed next to respective nodes. Analyses were rooted using the outer outgroup *Callihormius bifasciatus*. **A)** 50% completeness matrix, **B)** 70% completeness matrix.
