# Supplementary material for: Comprehensive phylogenomic analyses re-write the evolution of parasitism within cynipoid wasps
Source: BMC Evol Biol. 2020 Nov 23;20:155. doi: 10.1186/s12862-020-01716-2 (PMC7686688; doi:10.1186/s12862-020-01716-2)
Supplement: Supplementary file 12 — Additional file 12. Summary of phylogenetic positions for Eschatocerus niger. A) Eschatocerus niger grouping as sister to Figitidae, B) Eschatocerus niger grouping as sister to Cynipidae s.s., C) Eschatocerus niger grouping as sister to Figitidae + (Cynipidae s.s + (Diplolepidini + Pediaspidini)). Topology A is supported by all analyses on the full data set (all concatenated ML analyses and ASTRAL-III coalescent analysis) as well as four data subsets binned by GC content. Topology B is supported by four of the GC bins, while Topology C is supported by two of the GC bins. For details refer to Additional file 4. [file 12862_2020_1716_MOESM12_ESM.pdf]

***Additional files** for Blaimer et al: Comprehensive phylogenomic analyses re-write the evolution of parasitism within cynipoid wasps. BMC Evolutionary Biology.*

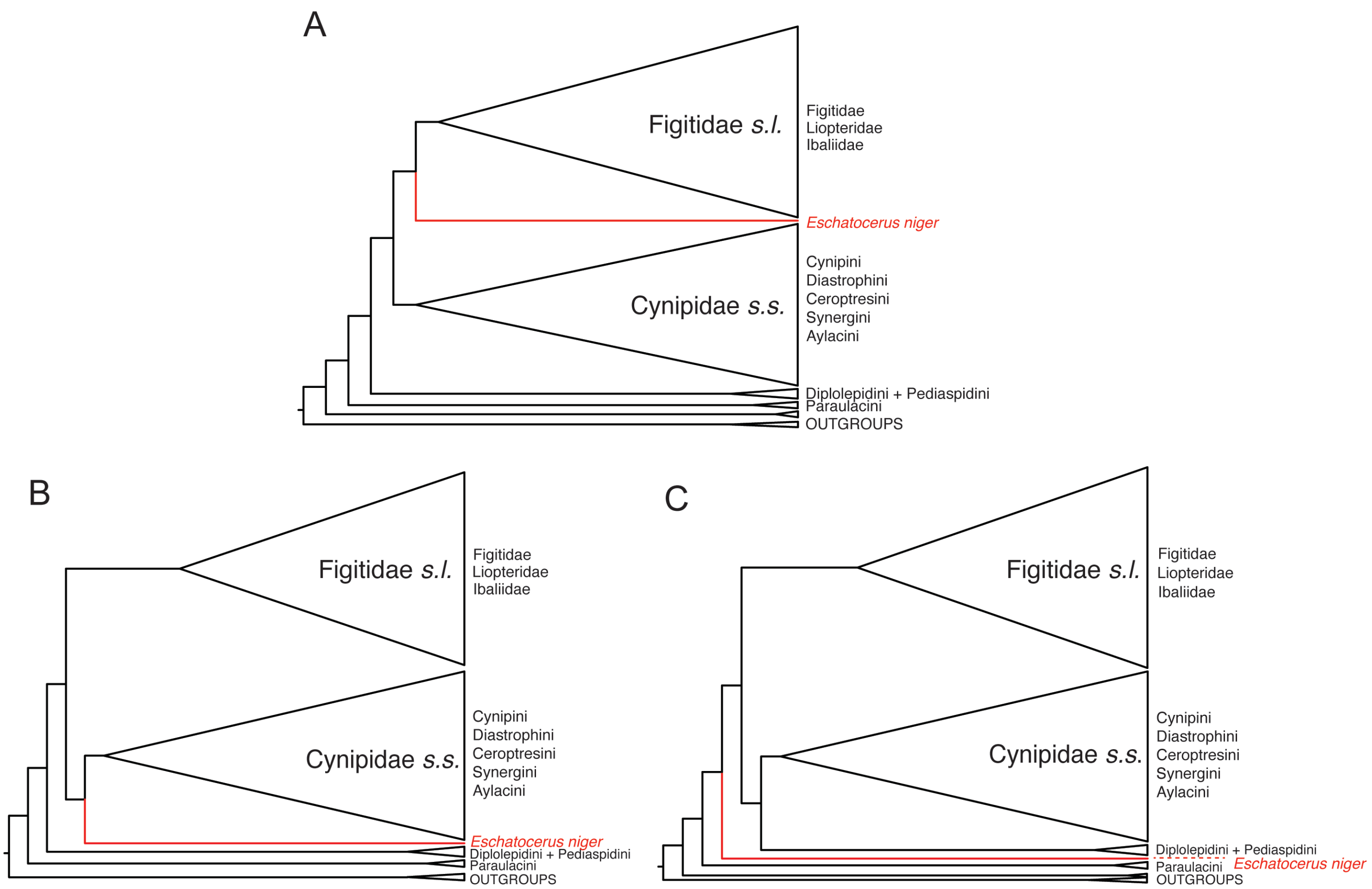


**Additional file 12: Summary of phylogenetic positions for *Eschatocerus niger*. A**) *Eschatocerus niger* grouping as sister to Figitidae, **B**) *Eschatocerus niger* grouping as sister to Cynipidae *s.s.*, **C**) *Eschatocerus niger* grouping as sister to Figitidae + (Cynipidae *s.s* + (Diplolepidini + Pediaspini)). Topology A is supported by all analyses on the full data set (all concatenated ML analyses and ASTRAL-III coalescent analysis) as well as four data subsets binned by GC content. Topology B is supported by four of the GC bins, while Topology C is supported by two of the GC bins. For details refer to Additional file 4.
